# SCRIB inhibits PP1 phosphatase activity without blocking or structurally altering its active site

**DOI:** 10.1016/j.jbc.2026.113599

**Published:** 2026-09-23

**Authors:** Siba Alharbi, Amit Kumawat, Brandon Huntington, Afaque A. Momin, Shaden Alfaiyz, Vincenzo di Florio, Marta Rigoli, Kelly Cardona Londono, Walter Rocchia, Anandsukeerthi Sandholu, Stefan T. Arold

**Affiliations:** 1 KAUST Center of Excellence for Smart Health, Biomedical Sciences Division, King Abdullah University of Science and Technology (KAUST), Thuwal, Kingdom of Saudi Arabia; 2 Department of Physics, University of Cagliari, Cagliari, Italy; 3 CONCEPT Lab, Istituto Italiano di Tecnologia, Genova, Italy; 4 MOX - Department of Mathematics, Politecnico di Milano, Milan, Italy

**Keywords:** cryo-EM, catalytic control, allostery, protein dynamics, entropy, variants of uncertain significance, latent pool hypothesis, molecular dynamics, paradoxical complex

## Abstract

Protein phosphorylation is a fundamental mechanism regulating cellular function, governed by the opposing actions of kinases and phosphatases. The substrate specificity and catalytic activity of protein phosphatase 1 (PP1), a major serine/threonine phosphatase, results from its interactions with more than 200 regulatory proteins. However, the mechanisms employed remain largely unknown. Here, we present the 3.5 Å cryo-electron microscopy structure of the complex between PP1 and the tumor suppressor and negative ERK-signaling regulator scribble homologue (SCRIB), revealing an unprecedented inhibitory mechanism. SCRIB engages PP1 *via* a multipronged interface composed of its leucine-rich repeat (LRR) domain, its first PDZ domain, and its flanking regions, where the ^705^KGVSF sequence acts as a non-canonical PP1-binding RVxF motif. Enzymatic kinetics demonstrate that SCRIB potently dampens catalytic turnover (*k*_*cat*_) while preserving PP1 in a fully ion-loaded, "active-like" state. Structural, computational, and evolutionary analyses suggest that SCRIB achieves this inhibition by allosterically modulating the electrostatic landscape and rigidifying the dynamic properties of the PP1 core. These findings identify SCRIB as the first known PP1 regulator that suppresses catalytic activity by conformationally locking an active-like state, rather than by disrupting the active site or occluding substrate-binding grooves. By sequestering PP1 in a primed yet silenced state, SCRIB establishes a latent cortical pool that provides a molecular mechanism for rapid, localized phosphatase activation following upstream stimuli.

Cellular function and survival depend on the precise regulation of protein activity. One of the most versatile and dynamic mechanisms for such regulation is protein phosphorylation, a reversible post-translational modification that controls protein activation, localization, interactions, and stability (1, 2, 3). This process is orchestrated by two opposing enzyme classes: kinases, which add phosphate groups, and phosphatases, which remove them. The correct balance between these opposing enzyme families is crucial for survival, as abnormally high or persistent phosphorylation is linked to diseases such as cancer, diabetes, inflammatory, neurodegenerative, and cardiovascular diseases (4, 5). Whereas kinases have been extensively studied and are well-characterized, protein phosphatases remain comparatively less understood, especially in terms of their regulatory mechanisms (5, 6).

Among these, protein phosphatase 1 (PP1) stands out as a central player (7). PP1 is responsible for more than half of all serine/threonine dephosphorylation events in eukaryotic cells and is involved in a wide range of biological processes, including glycogen metabolism, cell division, muscle contraction, neuronal signaling, and RNA splicing (8, 9). PP1 consists of only a catalytic core domain with short flanking regions (Fig. 1*A*). To ensure specific activity across its diverse functions, PP1 forms holoenzymes by associating with over 200 regulatory proteins (10). These interactions guide PP1 to its substrates and modulate its enzymatic function. Most regulatory proteins use a short linear motif as a primary PP1-binding motif, commonly referred to as the RVxF motif (where x is any residue). This motif, which has a hydrophobic core [V/I] x [F/W] preceded by one or more positive charges, associates with a largely hydrophobic surface pocket located more than 20 Å away from the PP1 active site (11, 12, 13). However, PP1 recognition mechanisms are diverse, and may involve other linear motifs or short alpha-helices (such as KiR and SILK) (14, 15, 16), and folded protein domains (such as SH3 and LRR) (17, 18, 19).

**Figure 1:**
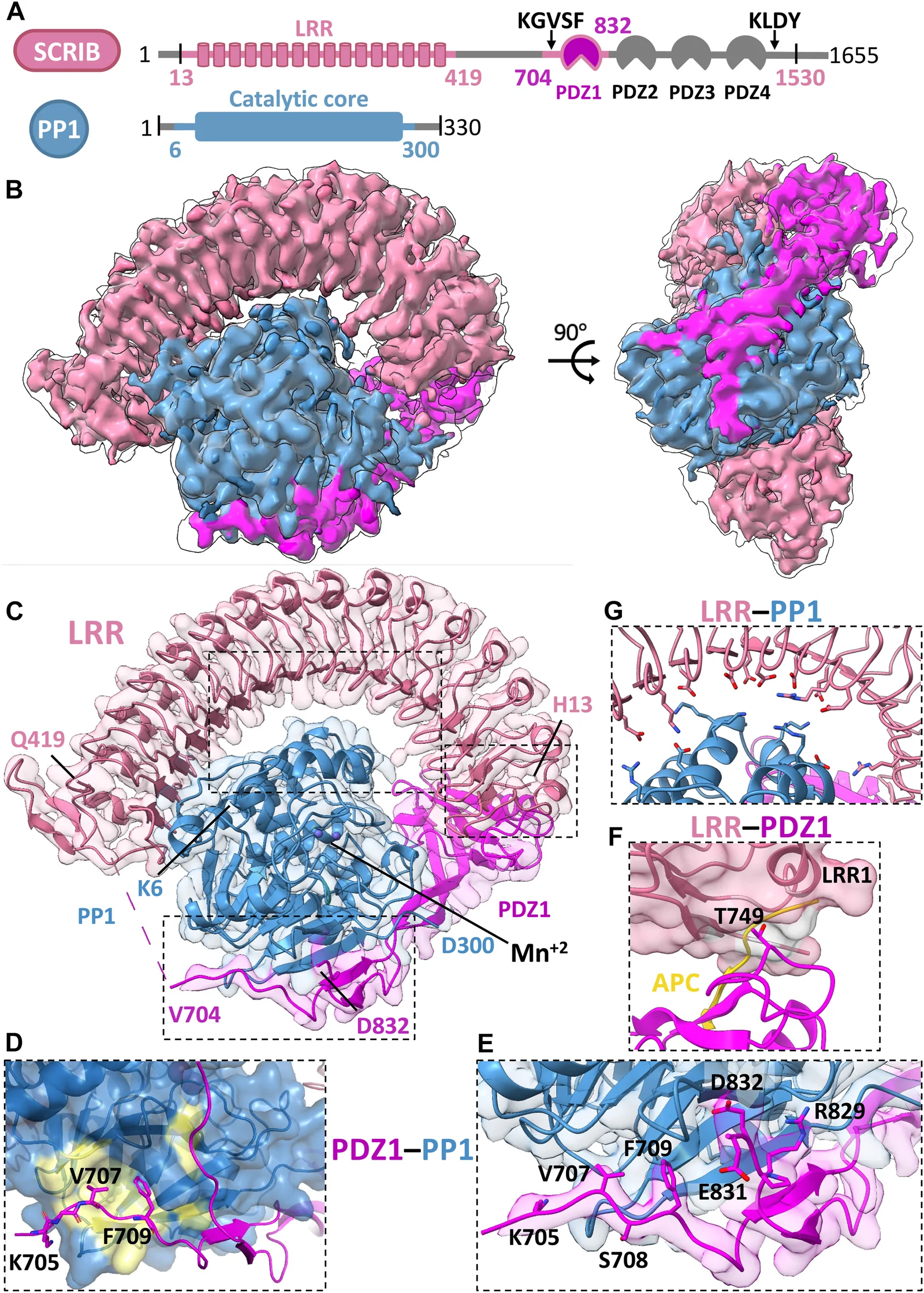
**Cryo-EM structure of the SCRIB–PP1 complex**. *A*, schematic representation of the domain architecture of SCRIB and PP1. Constructs used for structural determination of the complex are indicated with *black lines*. Regions resolved in the cryo-EM density are shown in *pink* and *deep purple* for SCRIB and *blu*e for PP1. *B*, cryo-EM reconstruction of SCRIB in complex with PP1. The unsharpened map is shown as *grey* transparent surface overlaid on the sharpened map, with domains colored as follows: PP1, *blue*; LRR, beige; PDZ1 and flanking regions, magenta. *C*, molecular structure of the SCRIB–PP1 complex. The EMReady map is shown as a colored transparent surface with the fitted atomic model. The catalytic core of PP1 is resolved with density in the active site consistent with two metal ions; two manganese ions are modeled to represent this dinuclear metal center. Key regions of the SCRIB–PP1 interface are highlighted. The residues shown indicate the extent of the modelled residues (K6–D300 for PP1; H13–Q419 for LRR; V704–D832 for the extended PDZ1). *D*, the RVxF pocket of PP1 engages the SCRIB KGVSF motif, with residues K705, V707, and F709 shown in stick representation. This interaction is reinforced by residues C-terminal to PDZ1. Shown is the molecular surface. Color coding as in (*C*) with yellow indicating the PP1 surfaces reported to bind the canonical RVxF motif. *E*, fit of the model to the sharpened EM map. *F*, PDZ1 docks into the hydrophobic pocket of the first LRR *via* T749. Superposed on the SCRIB–PP1 complex is the C-terminal adenomatous polyposis coli (APC) peptide (*yellow*, PDB 6MS1) to illustrate the competing interactions. *G*, the PP1–LRR interface. The interaction is mediated by polar and charged side chains.

A notable subset of PP1 regulatory proteins features leucine-rich repeat (LRR) domains, which facilitate the assembly of large, multi-domain holoenzymes (18, 20, 21, 22). One well-characterized example is SHOC2, an LRR-containing scaffold protein that forms a ternary complex with MRAS and PP1. This complex plays a pivotal role in the ERK1/2 MAPK signaling pathway, where it facilitates the dephosphorylation and activation of RAF kinases in response to extracellular signals. SHOC2 binds PP1 *via* a rudimentary RVxF motif (^64^VAF) and additional mostly electrostatic interactions mediated by its LRR domain, positioning PP1 for substrate access and enhancing its activity. Additionally, the SHOC2–PP1 association must be stabilized by GTP-bound MRAS at the membrane (23, 24, 25, 26). This mechanism is particularly relevant in the context of RAS-driven cancers, where SHOC2 has emerged as a potential therapeutic target (27, 28).

Conversely, the tumor suppressor Scribbled Homolog (SCRIB) opposes the action of SHOC2 by inhibiting PP1 and acting as a negative regulator of ERK signaling (21). SCRIB is a large, multi-domain protein containing an LRR domain and four PSD-95, Dlg, and ZO-1 (PDZ) domains (Fig. 1*A*). SCRIB interacts with PP1, SHOC2, and ERK at different stages of the signaling cascade (29, 30). Recent studies suggest that SCRIB competes with SHOC2 for PP1 binding, thereby inhibiting RAF dephosphorylation and dampening ERK pathway activation. However, the molecular mechanism by which SCRIB competes with SHOC2 and exerts this inhibitory effect on PP1 has remained elusive (21, 31, 32).

To address this gap, we determined the cryo-electron microscopy (cryo-EM) structure of the PP1–SCRIB complex. Our structure reveals that SCRIB engages PP1 through multiple interfaces: Its LRR domain forms weak electrostatic interactions that occlude one of PP1’s substrate-binding grooves, while its PDZ1 domain and an upstream modified RVxF motif (^705^KGVSF) further stabilize the interaction. Intriguingly, this complex is catalytically inactive, even though PP1 retains its active-site architecture and metal coordination. Our structural and biochemical analyses suggest that SCRIB inhibits PP1 through a combination of entropic and electrostatic mechanisms, effectively preventing substrate dephosphorylation despite an apparent active PP1 confirmation. These mechanistic insights into PP1 inhibition by SCRIB uncover an unusual mechanism of phosphatase regulation and advance our knowledge of ERK pathway modulation.

## Results and discussion

### Strategy for recombinant protein expression for *in vitro* analyses

AlphaFold predicts that the LRR and four PDZ domains of SCRIB are connected by long flexible linkers (see AF-Q14160-F1). Conversely, PP1 consists largely of a folded catalytic core, as first revealed in 1995 (33) (PDB 1FJM) (Fig. 1*A*). PP1 was efficiently expressed in *Escherichia coli* (see Experimental procedures). SCRIB LRR expression was reported only in insect cells (34). Attempts to express SCRIB alone in CHO or baculovirus-insect systems failed due to sustained protein degradation. To improve stability, we truncated SCRIB by 12 N-terminal and 125 C-terminal residues, predicted to be flexible and hence prone to degradation (Fig. 1*A*), and co-expressed SCRIB residues 13 to 1530 (henceforth referred to as SCRIB for simplicity) together with full-length PP1 using the pFastBac Dual vector in the Bac-to-Bac system. Size-exclusion chromatography (SEC) and SDS PAGE showed that this SCRIB–PP1 construct produced a stable complex suitable for cryo-EM analysis (Fig. S1*A*).

### SCRIB uses a multi-pronged association to capture PP1

We determined the 3.5 Å structure of the SCRIB–PP1 complex using single-particle cryo-electron microscopy (cryo-EM; Figs. S1*B*, S2, and Table S1; see Experimental procedures). The reconstruction clearly showed the characteristic horseshoe-shaped architecture of the LRR domain, partly encircling the PP1 catalytic domain (Fig. 1*B*). AlphaFold models for both domains could be fitted unambiguously into the cryo-EM density, supporting that the maps were not distorted by the slight directional resolution anisotropy of our data (Fig. S2*C*). Strong additional density extended from the N-terminal end of the LRR towards the final β-sheet of PP1 (magenta in Fig. 1*B*). Based on known PP1 structures (PDB 3EGG, 3HVQ), this final β-sheet can interact with RVxF peptides (35). SCRIB was suggested to harbor similar motifs upstream of PDZ1 (^705^KGVSF) and downstream of PDZ4 (^1275^KLDY) (21, 32). AlphaFold produced a model with good scores (ipTM = 0.86) and good fit to the residual density for the full KGVSF–PDZ1 fragment (residues 704–834) (Fig. S3*A*). Conversely, the PDZ4–KLDY fragment produced a lower AlphaFold score (ipTM = 0.7) and the predicted pose did not fit the density (Fig. S3*B*). Therefore, we modeled the complex based on the predicted structure of PP1 bound to SCRIB LRR and KGVSF–PDZ1.

The refined complex comprises the catalytic core of PP1 (residues 6–300), the SCRIB LRR (residues 13–419), which consists of 16 LRR repeats and is capped at its C-terminus by a short β-finger (residues 388–419), and the KGVSF–PDZ1 fragment (704–832) (Fig. 1*C*). Within this fragment, residues V707 and F709 of the KGVSF motif anchor into the PP1 hydrophobic groove, formed by β-strands β10, β11, β12, and β14. The adjacent residue K705 engages in an electrostatic interaction with PP1 residue D166 (Figs. 1, *D* and *E* and S3*C*). Thus, KGVSF mimics the canonical RVxF motif interaction observed in other PP1 regulators such as MYTP1 and the G_M_ subunit (36, 37). The linker region between the KGVSF motif and PDZ1 (residues 714–720) remains associated with PP1, contributing an additional β-strand to the complex (Figs. 1, *D* and *E* and S3*C*). Interestingly, the residues 818 to 832 following PDZ1 fold back onto the KGVSF–linker segment, reinforcing the interaction and forming additional contacts with strand β11 of PP1 (Figs. 1, *D* and *E* and S3*C*). Despite its inclusion in the complex, PDZ1 itself forms only minimal direct contacts with PP1, with PDZ1 residue T749 docking into a hydrophobic pocket formed by the first LRR. Structural comparisons with PDZ1 bound to C-terminal ligand peptides (*e.g.*, PDB 5VWI, 6MS1) reveal that engagement of these ligands would sterically clash with the PDZ1–LRR interface (38, 39) (Fig. 1*F*). This suggests that ligand binding to PDZ1 could disrupt its interaction with the LRR domain, potentially modulating PP1 association. Notably, the solvent-accessible surface buried between PP1 and the LRR domain is relatively small (1211.1 Å^2^) and mostly formed by long polar or charged side chains (Fig. 1, *C* and *G*).

We next performed binding studies to validate the overall architecture of our model. Isothermal titration calorimetry (ITC) revealed binding of PP1 to a 20-residue KGVSF motif–containing SCRIB peptide with a dissociation constant, *K*_*d*_, of 5.9 ± 1.2 μM. Conversely, no binding was detected for a peptide encompassing the KLDY motif (Fig. 2, *A* and *B*, Table 1, and Fig. S4*A*. See Experimental procedures for peptide sequences). ITC and microscale thermophoresis (MST) showed that the phenylalanine-to-alanine substitution of the KGVSF motif decreased the binding affinity by almost an order of magnitude (Fig. 2, *A* and *B*, Table 1, and Fig. S4*B*), in agreement with the importance of the phenylalanine in binding to the hydrophobic groove of PP1 (Fig. 1, *D* and *E* and Fig. S3*C*).

**Figure 2:**
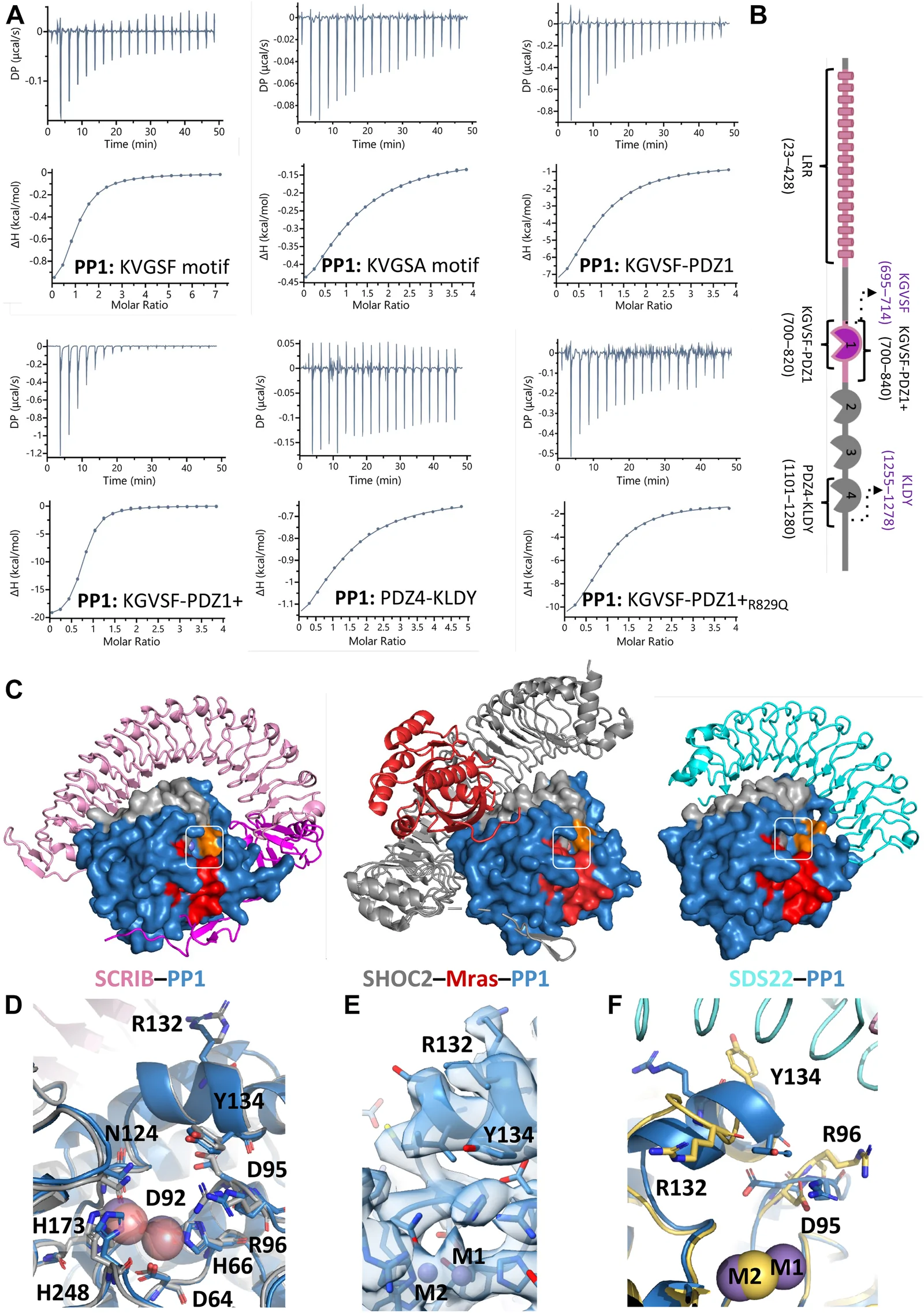
**SCRIB binding to PP1 leaves the active site solvent accessible**. *A*, representative isothermal titration calorimetry (ITC) interaction plots of the KGVSF and KGVSA motifs, KGVSF-PDZ1, KGVSF-PDZ1+, PDZ4-KLDY, and KGVSF-PDZ1+_R829Q_ titrated against PP1. All experiments were performed in triplicate. *B*, constructs used for biochemical analysis are mapped onto the schematic representation of the domain architecture of SCRIB. *C*, structural comparison between the SCRIB–PP1 complex, the SHOC2–MRAS–PP1 holoenzyme (PDB 7upi), and the SDS22–PP1 complex (PDB 6obn). Color-code is given in the complex composition below the structures. The surface of PP1 (*blue*) shows additionally the three binding areas, with *red*, *acidic groove*; *orange*, c-terminal groove, and *grey*, hydrophobic groove. The active site of PP1 is outlined in white. *D*, superimposition of the catalytic site of PP1 from the SCRIB–PP1 complex (PP1 in *blue*), and the SHOC2–MRAS–PP1 complex (PP1 in *gray*). *E*, fit between the cryo-EM map (*light blue*) and the PP1 model. Ion positions M1 and M2 are labelled. *F*, superimposition of the catalytic site of PP1 from the SCRIB–PP1 complex (PP1 in *blue*), and from the SDS22–PP1 complex (PDB 6obn, PP1 in *yellow*). Key PP1 residues remodelled by SDS22 are shown. Binding to SDS22 leads to the loss of ion M1 at the catalytic center. The remaining ion in M2 position is shown in yellow. Ions in both position in the SCRIB–PP1 complex are coloured in *magenta*.

**Table 1:** Summary of ITC Thermodynamic Parameters

Interaction (PP1)	MST	ITC				
	*K*_*d*_ (μM)	*K*_*d*_ (μM)	*N*	*ΔG* (kJ/mol)	ΔH (kJ/mol)	TΔS (kJ/mol)
KGVSF motif	2.8 ± 1.6	5.9 ± 1.2	1.0 ± 0.3	−29.9 ± 0.5	−4.6 ± 2.2	25.3 ± 2.6
KVGSA motif	60.7 ± 7.1	47.8 ± 3.5	1.0 ± 0.2	−24.4 ± 0.2	−2.5 ± 3.0	21.9 ± 5.0
KLDY motif	n.d.	–	–	–	–	–
KGVSF–PDZ1	n.d.	10.2 ± 0.4	0.9 ± 0.1	−28.5 ± 1.0	−46.6 ± 5.2	−18.1 ± 5.4
KGVSF–PDZ1+	2.5 ± 0.2	1.0 ± 0.1	0.8 ± 0.1	−34.3 ± 0.3	−79.4 ± 8.1	−45.2 ± 8.2
KGVSF–PDZ1+ _R829Q_	6.5 ± 2.1	3.8 ± 0.6	1.0 ± 0.1	−31.2 ± 0.8	−56.9 ± 4.7	−26.1 ± 5.1
PDZ4–KLDY	n.d.	19.9 ± 4.4	1.2 ± 0.2	−26.9 ± 0.5	−4.3 ± 0.4	22.6 ± 0.5
LRR	n.d.	–	–	–	–	–

Additional ITC profiles are provided in the Supplementary Material.

The KGVSF–PDZ1 fragment (residues 701–820) bound PP1 with an affinity similar to that of the KGVSF peptide alone (*K*_*d*_ = 10.2 ± 0.4 μM). In contrast, extending this construct to include the 17 C-terminal residues beyond PDZ1 that fold back onto the linker–KGVSF region in our model (KGVSF–PDZ1+, residues 701–840) increased binding affinity approximately tenfold (*K*_*d*_ = 1.0 ± 0.1 μM; Fig. 2, *A* and *B*, Table 1, Fig. S4*B*), confirming that the C-terminal region contributes to PP1 association. Conversely, the PDZ4–KLDY fragment bound PP1 only weakly (*K*_*d*_ ≈ 20 μM).

The isolated LRR domain (residues 23–428) failed to bind PP1 under our experimental conditions (Table 1 and Fig. S4*A*). This lack of detectable interaction is consistent with the limited contact surface observed between PP1 and the LRR in our structure and is further supported by unfavorable interaction parameters predicted by PDBePISA (ΔG = 1.2, ΔG *p*-value = 0.591). Additionally, the isolated PP1 domain required 500 mM NaCl for stability under the conditions used in ITC, a concentration that would weaken the predominantly electrostatic interactions seen in our structural model. Finally, cryo-EM analysis of LRR mixed with PP1 in a buffer containing 150 mM NaCl revealed several 2D class averages indicative of self-association between the concave surfaces of LRR molecules, (Fig. S5, *A* and *B*). This observation is consistent with previous reports showing that SCRIB’s LRR domain can homodimerize as a mechanism of autoinhibition (40), and it is therefore plausible that PP1 binding competes with this LRR self-association.

We were unable to determine the binding affinity between PP1 and full-length SCRIB, as the latter could not be purified and concentrated in its apo form. Taken together, our structural and biophysical data indicate that SCRIB engages PP1 through a combination of weak interactions involving its LRR and PDZ1 domains, as well as sequences flanking PDZ1. This multivalent interaction strategy may be critical for SCRIB’s inhibitory function toward PP1.

### SCRIB binding preserves PP1 in an active-like conformation

The active site of PP1 is at the intersection of three substrate-binding grooves, referred to as the hydrophobic groove, the acidic groove, and the C-terminal groove. These grooves can be recognized by docking motifs in regulatory proteins and/or position substrates for dephosphorylation (9, 14, 33, 41). In the SCRIB–PP1 complex, the C-terminal groove is partly occupied by the LRR and the KGVSF–PDZ1, while the LRR partially covers the hydrophobic surface adjacent to the hydrophobic groove (Fig. 2*C*). In contrast, the acidic groove and the active site of PP1 (residues Asp64, His66, Asp92, Asn124, His173, and His248) remain solvent accessible.

Intriguingly, when bound to SCRIB, PP1 adopts a virtually identical conformation as in the active SHOC2–MRAS–PP1 holoenzyme (C^α^ RMSD = 0.7 Å for 293 residues). Therefore, we refer to the PP1 conformation in the SCRIB complex as ‘active-like’. The cryo-EM density further supports a catalytically competent active site, with all metal-coordinating residues correctly positioned: Asp64 and His66 to coordinate metal ion in site 1 (M1), Asn124, His173, and His248 to coordinate the metal ion in site 2 (M2), and Asp92 bridging both ions. Additionally, Tyr134, positioned within the β12–β13 loop, engages Arg96 in the β/α loop near the active site through a combination of π-stacking and hydrogen bonding (Fig. 2, *D* and *E*). This dual interaction reinforces the metal-binding environment and promotes accurate substrate positioning and transition state stabilization, thereby ensuring optimal coordination of the catalytic metals and precise alignment of the scissile phosphate for efficient dephosphorylation (12, 18, 22, 33, 42).

Consistent with the active-like position of the metal-coordinating side chains, the cryo-EM density in the active site supports the presence of both ions (in M1 and M2 positions), although the resolution is insufficient to unambiguously identify them (Fig. 2*E*). Given that previous studies have detected Mn^2+^ ions at these positions in PP1 purified from SF9 cells (24), and considering that manganese was present in both the culture medium and lysis buffer (s. Experimental procedures), we modelled two Mn^2+^ ions in this density. However, the insect cell medium also contained zinc and iron and we cannot exclude the possibility of partial occupancy by these alternative ions.

Conversely, the cryo-EM density for PP1 in its complex with SCRIB does not support the inactivating mechanism employed by SDS22, which uses its LRR domain to bind to the C-terminal groove and, in so doing, restructures two loops (residues 95–99 and 127–135). This restructuring leads to the loss of the M1 ion and inactivates PP1 (18, 22) (PDB 6OBN) (Fig. 2, *C* and *F*). Although the LRRs of SDS22 and SCRIB are positioned similarly on PP1, SCRIB does not reorient these loops and preserves the catalytic site of PP1 in an apparently active conformation.

### SCRIB binding inhibits the catalytic activity of PP1

The observation that SCRIB captures PP1 in an apparently active conformation is surprising, given that SCRIB is reported to be an inhibitor of PP1 (21, 29, 32, 43). Although the inhibitory action of SCRIB on PP1 has been demonstrated in a cellular context, it has not yet been shown for the isolated recombinant SCRIB–PP1 complex.

We first assessed the effect of SCRIB on PP1 using the malachite green assay with a natural substrate mimic, a synthetic peptide representing Ser365–phosphorylated BRAF residues 358 to 370 (pBRAF). This assay was performed with a buffer containing 2 mM MnCl_2_ to assure a full occupation of PP1 with manganese ions. PP1 alone and PP1 N-terminally fused to maltose binding protein (MBP–PP1) efficiently dephosphorylated pBRAF and their dephosphorylation activity correlated with protein concentration (Fig. 3*A*). PP1 pre-incubated with individual SCRIB fragments (KGVSF–PDZ1, LRR, or both) also dephosphorylated pBRAF (Fig. 3, *A*–*C*). In fact, the presence of KGVSF–PDZ1 and/or LRR marginally increased the activity of PP1, though this effect was generally not statistically significant. The MBP fusion similarly produced a slight activity enhancement of PP1 compared to PP1 without MBP. These findings are consistent with a low affinity of the isolated KGVSF–PDZ1 and LRR domains toward PP1 (below the detection limit in our analyses; Fig. S4*A*), suggesting that the modest activity enhancement may result from transient stabilizing interactions rather than specific regulatory effects.

**Figure 3:**
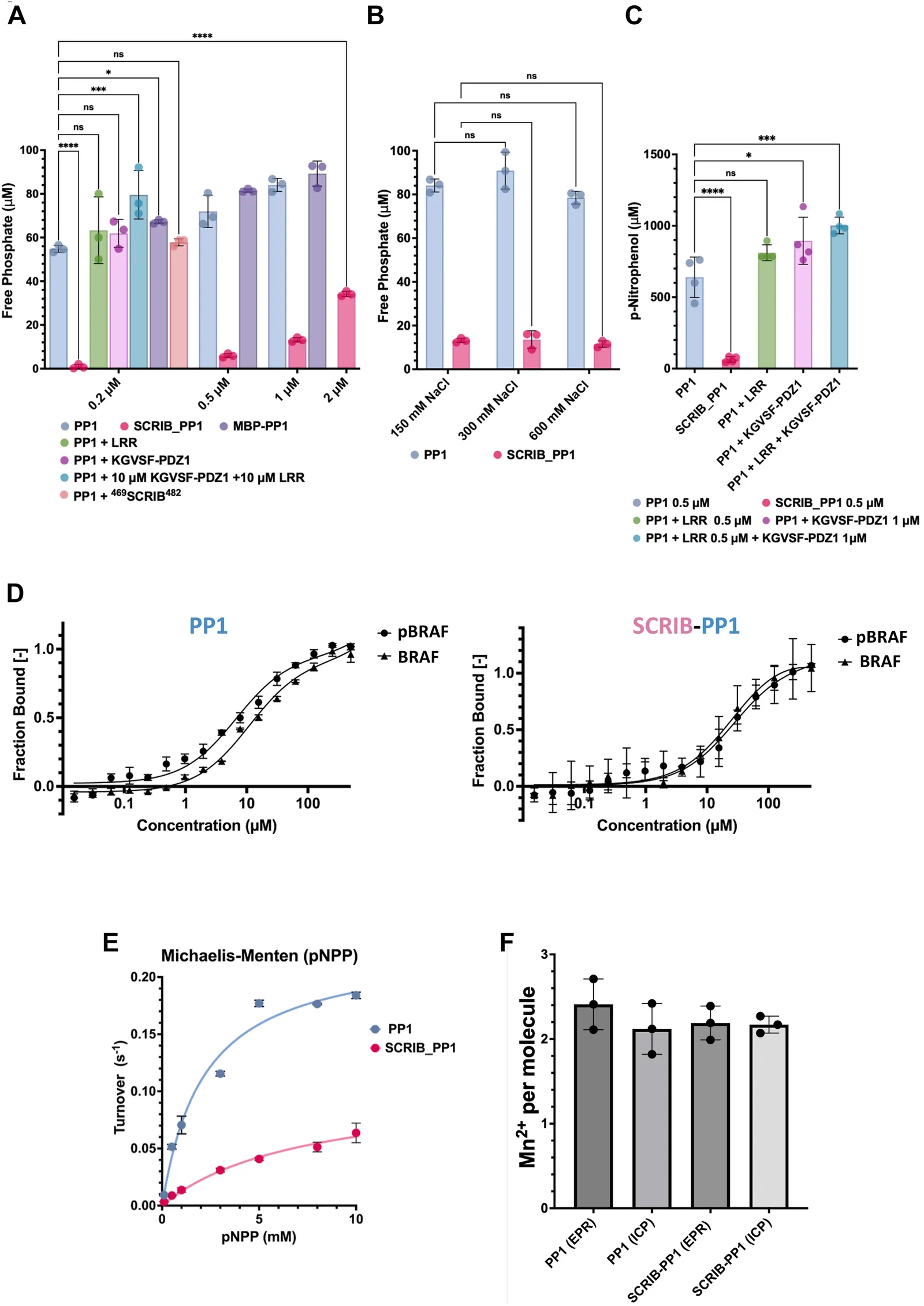
**SCRIB binding inhibits PP1 dephosphorylation activity.***A*, dephosphorylation of a phosphorylated BRAF peptide by PP1 under different conditions. PP1 alone, PP1 pre-incubated with individual SCRIB fragments (KGVSF–PDZ1, LRR, or both), MBP-tagged PP1, and PP1 saturated with a synthetic SCRIB peptide (residues 469–482) efficiently dephosphorylate pBRAF. In contrast, the co-purified SCRIB–PP1 complex exhibits a marked reduction in catalytic activity, even at higher PP1 concentrations of 0.5, 1, and 2 μM. Error bars represent mean ± SD of triplicate measurements. *B*, increasing salt concentrations had no effect on PP1 activity or the reduced activity of the SCRIB–PP1 complex, indicating the inhibition is not driven by simple electrostatics. *C*, SCRIB binding inhibits PP1 activity toward small-molecule substrates. PP1, or PP1 pre-incubation with SCRIB fragments, efficiently dephosphorylated pNPP, whereas the co-purified SCRIB–PP1 complex retained only residual activity. Error bars represent mean ± SD of four replicates. Statistical significance was assessed by one-way ANOVA with multiple-comparisons testing. Significance is indicated as follows: ns, not significant, *p* ≥ 0.05; (∗) *p* < 0.05; (∗∗∗) *p* < 0.001; (∗∗∗∗) *p* < 0.0001. *D*, binding curves for pBRAF and BRAF to PP1 and SCRIB-PP1 determined by MST. Increasing concentrations of each peptide (BRAF and pBRAF) were titrated against PP1 or SCRIB-PP1, and the fraction bound was fitted to a one-site binding to extract apparent binding affinity. Error bars represent mean ± SD from independent measurements. *E*, representative pNPP dephosphorylation assay. PP1 shows robust catalytic activity, whereas the SCRIB–PP1 complex displays markedly reduced efficiency. Results are representative of two independent experiments over 30 min. *F*, Mn^2+^ stoichiometry per protein molecule for recombinant PP1 and SCRIB-PP1 measured by continuous-wave EPR spin counting and ICP-MS. For EPR, stoichiometry was obtained by double integration of the Mn^2+^ signal relative to an external Mn^2+^ standard; error bars represent the standard deviation of 3 independent measurements. For ICP-MS, stoichiometry was calculated from the background-corrected Mn^2+^ concentration and the protein concentration determined by A280; error bars represent propagated uncertainty from calibration, background subtraction, and protein concentration errors combined in quadrature.

Conversely, the co-purified SCRIB–PP1 complex exhibited a substantially reduced pBRAF dephosphorylation activity (Fig. 3*A*). Even at a concentration of 2 μM SCRIB–PP1 exhibited a substantially lower activity than apo-PP1 at 0.2 μM, ruling out that the inhibitory effect is caused by dilution effects or by overestimated PP1 protein concentrations due to the presence of unliganded apo-SCRIB. Finally, the addition of salt (to a final NaCl concentration of 300 or 600 mM) did not affect the activities of PP1 and SCRIB–PP1, indicating that long-range electrostatic effects did not strongly influence these reactions (Fig. 3*B*). We concluded that SCRIB strongly inhibits dephosphorylation of BRAF by PP1 *in vitro*.

### Inhibition of PP1 is not explained by SCRIB blocking substrate interactions

Our cryo-EM analysis revealed that SCRIB contacts regions of the C-terminal and hydrophobic substrate-binding grooves of PP1. In principle, the inhibition of pBRAF dephosphorylation by SCRIB could therefore arise from partial occlusion of these grooves. A similar mechanism has been described for the neuronal PP1 regulator spinophilin, which also contains a PDZ domain. Spinophilin maintains PP1 in an active conformation (PDB 3EGG; RMSD to PP1 in the SCRIB–PP1 complex = 0.5 Å over 294 residues) but alters substrate specificity by restricting access to the C-terminal groove (35). As a consequence, spinophilin-bound PP1 is unable to dephosphorylate phosphorylase *a*, a substrate that relies on this groove for binding.

To determine whether SCRIB inhibits PP1 by restricting substrate binding, we measured the affinity of BRAF-derived peptides for PP1. Mn^2+^-bound PP1 exhibited a slight preference for phosphorylated pBRAF over the corresponding unphosphorylated peptide (*K*_*d*_ = 6.63 ± 1.01 μM and 10.9 ± 1.8 μM, respectively) (Fig. 3*D*). Chelation of the catalytic metal ions with EGTA reduced the affinity for pBRAF approximately twofold (*K*_*d*_ = 12.7 ± 2.0 μM), consistent with a modest but measurable contribution of the phosphate group and catalytic metal center to substrate recognition (Fig. S4*C*).

Both BRAF and pBRAF peptides remained capable of binding the SCRIB–PP1 complex. However, in the presence of SCRIB, phosphorylated and unphosphorylated peptides bound with similar affinities (*K*_*d*_ = 27.1 ± 2.5 and 26.2 ± 2.6 μM, respectively), corresponding to an approximately threefold reduction relative to PP1 alone (Fig. 3*D*). The loss of preferential binding to the phosphorylated peptide indicates that SCRIB diminishes the energetic contribution of the phosphate moiety to substrate recognition. Nevertheless, this effect is modest. Given that pBRAF was present at 100 μM in the dephosphorylation assays, a threefold reduction in substrate affinity cannot account for the near-complete inhibition of PP1 activity by SCRIB (Fig. 3*A*). These findings therefore argue against a mechanism in which SCRIB primarily inhibits PP1 by blocking substrate engagement at the C-terminal or hydrophobic grooves. Instead, SCRIB appears to modestly weaken phosphate-dependent substrate recognition while exerting its principal inhibitory effect at a subsequent step in the catalytic cycle.

### SCRIB does not directly occlude the active site of PP1

Although SCRIB did not prevent BRAF binding to PP1, it remained possible that SCRIB permits substrate association while obstructing access of the phosphoserine residue to the catalytic center. Indeed, several PP1 inhibitors act through direct occlusion of the catalytic center, including inhibitor-3 (PDB 8DWL) (44) and the natural toxins microcystin-LR (PDB 1FJM) (33), calyculin A (PDB 1IT6) (45), nodularin-R (PDB 3E7A), and tautomycin (PDB 3E7B) (46). Consistent with such a mechanism, AlphaFold models of the SCRIB–PP1 complex suggested that the linker connecting the LRR and KGVSF–PDZ1 regions (residues 469–482) binds directly to the PP1 active site (Fig. S6*A*). However, a synthetic peptide corresponding to SCRIB residues 469 to 482 failed to inhibit PP1 activity even at saturating concentrations (Fig. 3*A*). Furthermore, inspection of cryo-EM maps at lower contour levels, as well as B-factor-blurred reconstructions, revealed no residual density within the catalytic cleft that would indicate direct occupation of the active site by SCRIB (Fig. S6*A*).

To further rule out that SCRIB occludes the active site accession of substrates, we examined the ability of PP1 to dephosphorylate para-nitrophenyl phosphate (pNPP), a small generic phosphatase substrate that interacts only with the active site and does not require auxiliary substrate-binding grooves. Recombinant PP1 efficiently hydrolyzed pNPP, and this activity was retained when PP1 was pre-incubated with individual SCRIB fragments (KGVSF–PDZ1, LRR, or both; Fig. 3*C*). As observed in our dephosphorylation assays with pBRAF, the addition of KGVSF–PDZ1 and/or LRR resulted in a modest and not always statistically significant increase in PP1 activity. In contrast, the purified SCRIB–PP1 complex displayed only residual pNPP phosphatase activity (Fig. 3*C*) and even 2 μM SCRIB–PP1 complex exhibited substantially lower activity than 0.2 μM PP1 alone (Fig. S6*B*).

To quantify the effect of SCRIB on PP1 catalysis, we performed Michaelis–Menten kinetic analyses using pNPP as substrate (Fig. 3*E* and Table 2). Binding of SCRIB reduced the apparent substrate affinity of PP1 by approximately threefold (*K*_*m*_ = 7.3 mM *vs* 2.2 mM for PP1 alone), but the effect on catalytic turnover was far more pronounced, with the turnover number decreasing by more than twentyfold upon SCRIB binding (*k*_*cat*_ = 1.1 s−^1^ *vs* 0.05 s−^1^ for PP1). Thus, while SCRIB modestly impairs substrate binding, its inhibitory effect is dominated by a profound reduction in catalytic efficiency.

**Table 2:** Summary table of Michaelis–Menten Kinetic Parameters

Protein	PP1	SCRIB-PP1
*E*_*t*_	0.2 μM	2 μM
*V*_*max*_	0.20 ± 0.02 μM s^−1^	0.10 ± 0.02 μM s^−1^
*K*_*m*_	2.2 ± 0.4 mM	7.3 ± 2.6 mM
*k*_*cat*_	1.0 ± 0.1 s^−1^	0.05 ± 0.01 s^−1^
*k*_*cat*_*/K*_*m*_	0.50 mM^−1^s^−1^	0.01 mM^−1^s^−^

Enzymatic constants derived from pNPP assays for PP1 and the SCRIB–PP1 complex.

Collectively, these results demonstrate that SCRIB inhibits PP1 through a mechanism that cannot be explained by steric blockage of substrate recruitment or direct occlusion of the catalytic center. Instead, SCRIB primarily suppresses the catalytic activity of PP1 after substrate binding has occurred.

### SCRIB does not impede the coordination of metal ions by PP1

Although the cryo-EM density did not support an SDS22-like inhibitory mechanism involving loop remodeling and release of a catalytic metal ion, the moderate resolution of the cryo-EM maps warranted independent verification that both catalytic metal ions remain bound in the SCRIB–PP1 complex.

We first confirmed that SCRIB inhibits PP1 phosphatase activity to an extent comparable to either manganese depletion by EGTA (10 mM) or inhibition by the phosphatase inhibitor NaF (10 mM) (Fig. 3*F*). However, MST demonstrated that Mn^2+^ binds to PP1 and the SCRIB–PP1 complex with similar affinities of approximately 20 μM (Fig. S4*D*). In contrast, control MST experiments showed no detectable binding of Ca^2+^ to either PP1 or SCRIB–PP1 and no binding of Mn^2+^ to lysozyme, indicating that the observed interaction is specific (Fig. S4*E*).

To directly determine the number of Mn^2+^ ions associated with PP1 in the absence and presence of SCRIB, we performed room-temperature X-band electron paramagnetic resonance (EPR) spectroscopy. To minimize contributions from non-specifically bound manganese, all samples were dialyzed extensively against Mn^2+^-free buffer before analysis. Free Mn^2+^ produced the expected EPR signal, whereas the dialyzed lysozyme control retained only 10.1 μM Mn^2+^, establishing the background level of non-specific metal association. In contrast, PP1 (27.5 μM protein) contained 76.7 μM total Mn^2+^ (66.6 μM after background subtraction), whereas the SCRIB–PP1 complex (7.5 μM protein) contained 26.5 μM total Mn^2+^ (16.5 μM net Mn^2+^). Normalization to protein concentration yielded 2.4 ± 0.3 Mn^2+^ ions per PP1 molecule and 2.2 ± 0.2 Mn^2+^ ions per SCRIB–PP1 complex (Figs. 3*F*, S7, *A* and *B*, and Table S2). These values indicate that SCRIB binding does not affect metal loading and that both PP1 alone and the SCRIB-bound complex retain full occupancy of the two catalytic Mn^2+^ sites.

As an orthogonal validation, we quantified metal content by inductively coupled plasma mass spectrometry (ICP-MS), a method previously used to assess PP1 metal coordination (33, 47, 48). ICP-MS measurements yielded net Mn^2+^ concentrations of 52.6 ± 2.1 μM for PP1 (27.5 μM protein) and 16.0 ± 1.2 μM for the SCRIB–PP1 complex (7.5 μM protein). These values correspond to stoichiometries of 2.1 ± 0.3 Mn^2+^ ions per PP1 molecule and 2.2 ± 0.2 Mn^2+^ ions per SCRIB–PP1 complex, respectively (Fig. 3*F*, ICP-MS bars).

Together, the MST, EPR, and ICP-MS data demonstrate that PP1 retains two catalytic Mn^2+^ ions in the presence of SCRIB. Thus, SCRIB-mediated inhibition does not arise from disruption of metal coordination or depletion of catalytic metals, indicating that its inhibitory mechanism is fundamentally distinct from metal-ejection mechanisms described for SDS22 (see above) and Inhibitor-2, which positions helical elements across the PP1 active site that additionally displace at least one of the metal ions through a tyrosine that reaches into the active site (PDB 2O8G) (49).

### SCRIB LRR modifies the electrostatic environment around the active site

Having established that SCRIB-mediated inhibition of PP1 does not arise from major conformational changes, active-site occlusion, or loss of catalytic metal ions, we next considered long-range electrostatic and allosteric mechanisms. Mapping the vacuum electrostatics onto the surface of the SCRIB–PP1 complex revealed that the SCRIB LRR is highly electronegative at its interface with PP1 (Fig. 4, *A* and *B*). The total negative charge of the concave LRR surface is conserved across SCRIB homologues from humans to worms, whereas a putative bacterial ancestor of the LRR domain (the leucine-rich repeat protein from *Leptospira interrogans*) (50, 51, 52) lacks this signature (Fig. S8). Moreover, the LRRs of spinophilin and SHOC2 have a strongly positive patch close to the PP1 active site (Fig. 4, *C* and *D*), suggesting the charge distribution of SCRIB is relevant for PP1 inhibition. The electronegativity of the SCRIB LRR is jointly produced by a large number of negatively charged amino acids (a total of 23 aspartic acids and 34 glutamic acids). Many of these acidic residues are stabilizing the LRR domain and/or its interaction with SCRIB, making it impracticable to test their effect by mutational analysis. We therefore probed the electrostatic influence of SCRIB on PP1 computationally.

**Figure 4:**
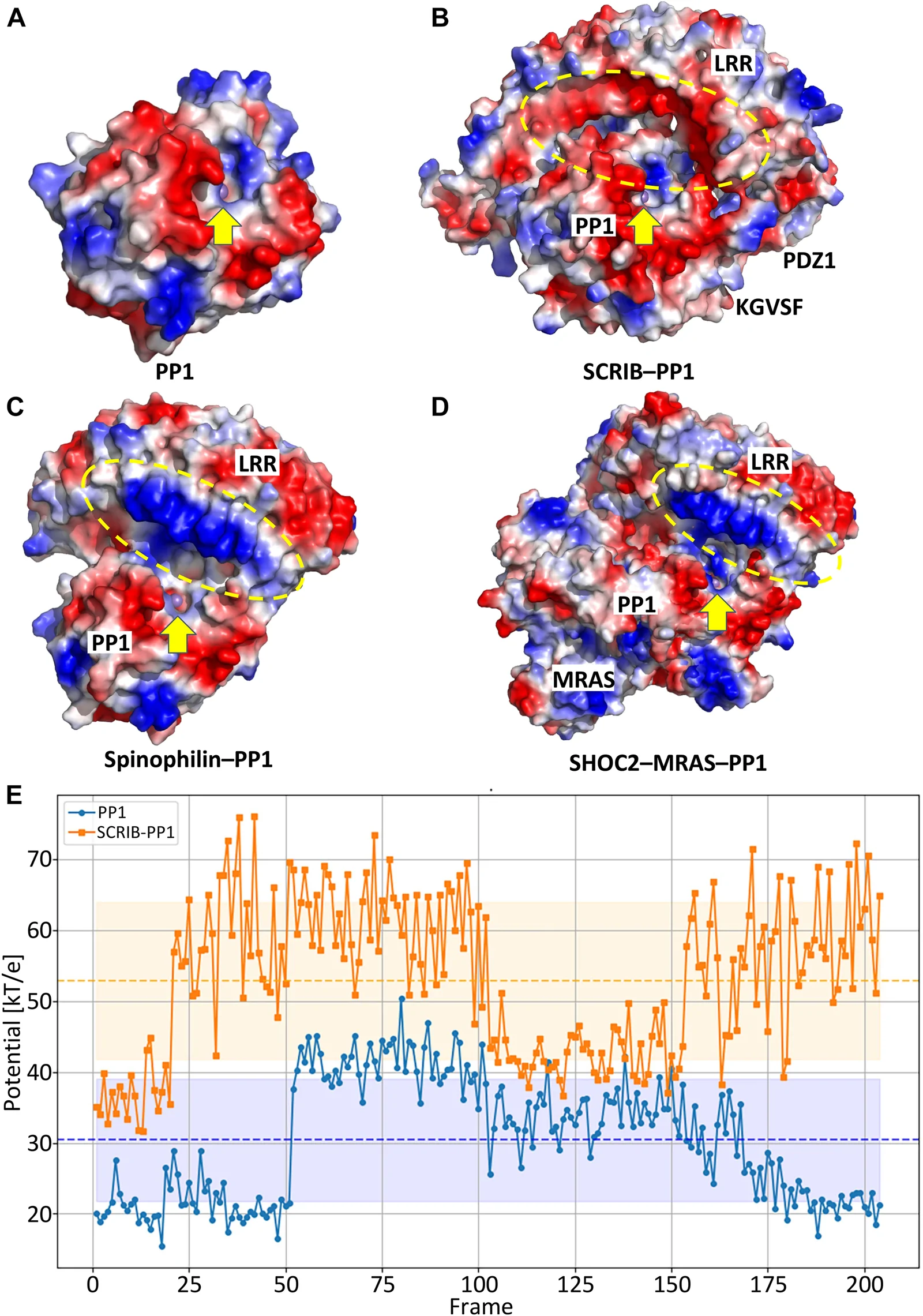
**Electrostatic and solvent-exposure characterization of the SCRIB–PP1 complex.***A–D*, Vacuum electrostatics mapped onto PP1 complexes show that the SCRIB LRR interface is strongly electronegative compared with other PP1 regulators. *A*, PP1 alone (PDB 1IT6). *B*, SCRIB–PP1. *C*, Spinophilin–PP1 (PDB 3EGG). *D*, SHOC2–MRAS–PP1 (PDB 7UPI). *E*, electrostatic potential variation at the PP1 active site. Continuum electrostatics calculations were performed over 200 frames extracted from 4 independent molecular dynamics trajectories modeling the interaction of PP1 and PP1-SCRIB with the target peptide. To make comparable the potential value estimates extracted from different trajectories, we decided to trace the position of the (de)phosphorylation site along them. Therefore, we removed the target peptide from both trajectories and evaluated the electrostatic potential at its position. This analysis shows an increase in the local potential when SCRIB is present. This increase is not due directly to the potential generated by SCRIB, which is overall negative, but an indirect adaptation of PP1 to the SCRIB presence. For sake of completeness, we monitored the distance of our probe location from the nearby Mn^2+^ ions, which turned out to be quite conserved, dist(P,Mn1) ∊ [2.91 Å; 3.61 Å], dist(P,Mn2) ∊ [3.39 Å; 3.90 Å] in the PP1 trajectory, dist(P,Mn1) ∊ [2.98 Å; 3.59 Å], and dist(P,Mn2) ∊ [3.39 Å; 3.96 Å] in the PP1-SCRIB trajectory, respectively.

We first calculated the interaction of the pBRAF target peptide with either PP1 alone or the SCRIB–PP1 complex from the molecular dynamics (MD) simulation trajectories. We then removed pBRAF from the simulation trajectories and only retained the coordinates of the phosphorus atom of pSer365 (see Experimental procedures and Fig. 4*E*). Continuum electrostatics calculated for this location showed, unexpectedly, that the potential at the phosphorus position is more positive along the trajectories when SCRIB is present. Thus, although the electrostatic impact of SCRIB overall negativizes the PP1 surface around the active site, it produces a localized opposite effect in proximity to the catalytic center. This seems to be due to an indirect effect of SCRIB on PP1, which could cause the positive change in the local potential. These calculations supported that the major inhibitory effect of SCRIB is not due to electrostatic repulsion of the target peptide. This conclusion is in line with our observations that high salt does not affect dephosphorylation, and that the presence of SCRIB only mildly reduced the substrate affinity *k*_*m*_ of PP1 (Fig. 3, *B* and *E* and Table 2). However, the changes in the electrostatic potential at the PP1 active site, which includes several titratable residues and two Mn^2+^ ions, may negatively affect the dephosphorylation reaction, and thereby contribute to the observed reduction in substrate turnover (*k*_*cat*_; Fig. 3*E* and Table 2).

### SCRIB binding rigidifies conformational dynamics of PP1

Although PP1 adapted an active-like structure in the complex with SCRIB, we reasoned that the inhibitory action of SCRIB could stem from subtle alterations of the structural dynamics of PP1. We performed four 1-μs MD simulations of PP1 in both its apo form and in complex with the LRR and KGVSF–PDZ1 fragments of SCRIB, based on our cryo-EM structural model. All simulation replicates remained structurally stable throughout (Fig. S9, *A* and *B*). Residue-wise root mean square fluctuation (RMSF) analysis of the MD simulation data revealed markedly reduced flexibility for several regions upon SCRIB binding (Fig. 5*A*). These regions were directly or indirectly in contact with SCRIB: The PP1 loop comprising residues 18 to 25 (forming one side of the C-terminal groove) abuts the PDZ1 domain. The helix formed by residues 129 to 135 next to the active site (which was remodeled by the SDS22 LRR domain but not by SCRIB) is in electrostatic contact (through Arg132) with the SCRIB LRR; residue Arg221 coordinating the phosphate above the M1 ion position; the active-site proximal loop 270 to 280, which is connected to the KGVSF–PDZ1 linker through the PP1 C-terminal tail (residues 296–303, also stabilized in the complex); and residues 286 to 291 that bind to the KGVSF motif. The RMSF reductions of the residues surrounding the active site (129–135, 221, and 270–280) were of particular interest, as they indicate a restriction of active-site fluctuations. For example, dihedral sampling of Tyr134 showed multiple χ_1_/χ_2_ rotamers in apo-PP1, whereas this distribution narrowed significantly in the SCRIB-bound complex, indicating constrained side-chain mobility (Fig. S9, *C* and *D*).

**Figure 5:**
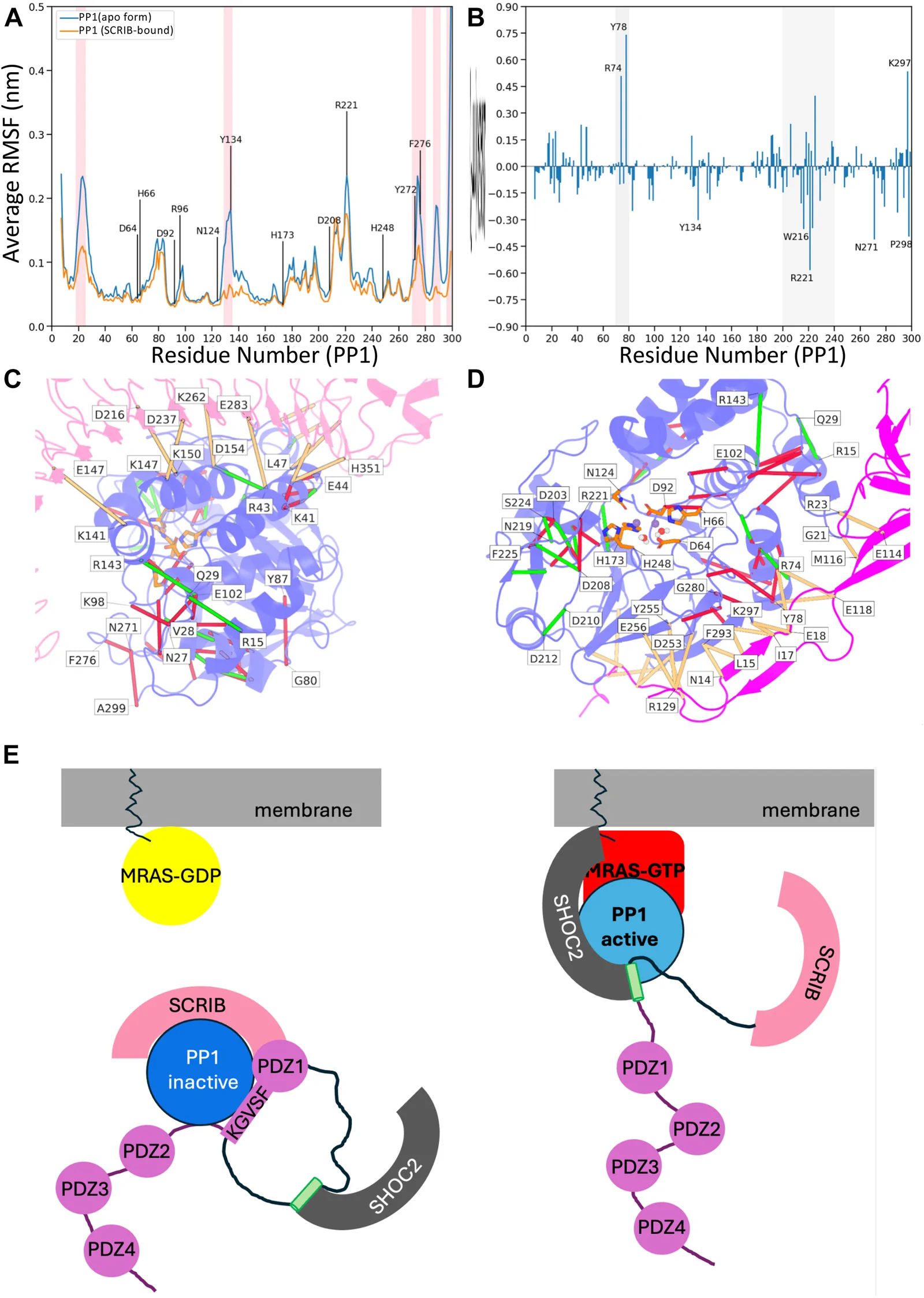
**SCRIB binding modulates PP1 dynamics, solvent accessibility, and hydrogen-bond networks, suggesting a model for PP1 regulation.***A* and *B*, backbone root mean square fluctuation (RMSF) and residue-wise solvent accessibility changes of PP1 in the apo form and in complex with LRR and PDZ1 domains of SCRIB. (*A*) RMSF values for PP1 residues averaged over all four replicas for each system. Notable differences in flexibility are observed in specific regions (residues 18–25, 130–141, and 270–290), highlighting reduced fluctuations in the complex and suggesting stabilization upon SCRIB binding. *B*, for each PP1 residue, solvent-accessible surface area (SASA) was averaged over all four replicas, and the difference was calculated as. Bars above zero indicate increased exposure and bars below zero indicate burial upon binding. Shaded bands mark contiguous segments showing coordinated changes. *C* and *D*, hydrogen-bond rearrangement upon formation of the PP1–LRR-PDZ1 complex. PP1, LRR, and PDZ1 are shown as cartoon representations coloured *skyblue*, *pink*, and *magenta*, respectively. H-bonds formed exclusively at the (*C*) PP1–LRR and (*D*) PP1–PDZ1 interfaces are depicted as orange sticks. Changes in intramolecular PP1 H-bond occupancy between apo and bound states are mapped onto the PP1 structure as net H-bond occupancy (%). *Green lines* represent hydrogen bonds with higher occupancy (>20%) in the apo form (lost upon complex formation), whereas *red lines* represent hydrogen bonds with higher occupancy (>20%) in the complex (gained upon binding). *E*, proposed model of PP1 regulation. In the absence of GTP–MRAS, SCRIB sequesters and inhibits PP1 while remaining associated with SHOC2. Upon MRAS activation, the SHOC2–SCRIB–PP1 complex is recruited to the membrane, where MRAS displaces SCRIB and promotes SHOC2–PP1 association, enabling PP1 activation. The bold font indicates MRAS and PP1 in their active form.

We further quantified solvent accessibility of PP1 residues during the MD simulations, in the presence and absence of SCRIB (Fig. 5*B*). SCRIB binding reduced the solvent exposure of PP1 residues R221 and Y134 by 58% and 30%, respectively. Arg96, which co-coordinates the substrate phosphate with Arg221 (33), did not show altered RMSF or SASA in the SCRIB complex but engages in cation–π interactions with Tyr134. Notably, the minimum distance distributions of the Y134–R96 and D208–R221 residue pairs collapsed into narrow, short-distance modes in the complex, compared to broader distributions in the apo state (Fig. S10, *A*–*C*). Additionally, SCRIB binding was computationally associated with a modest but consistent reduction in local water density, and a narrowing of the radius of gyration around the catalytic Mn^2+^ ions, further supporting a stabilization of the active site environment (Fig. S10, *D*–*F*).

An analysis of residue–residue contact maps and hydrogen bonding networks between apo-PP1 and the complexed form provided a rationale for the altered dynamics: We observed that the formation of interfacial contacts with SCRIB triggers a reorganization of the intramolecular interaction network of PP1 (Fig. S11, *A* and *B*). The resulting net increase in hydrogen bond number and occupancy reduces the conformational entropy of PP1 (Fig. 5, *C* and *D*), and confines it to a more rigid, low-entropy state. RMSF calculations from MD simulation trajectories (Fig. 5*A*) and B-factor coloring of the cryo-EM structure (Fig. S12) supported that the LRR, and to a lesser extent the KGVSF region, rigidify PP1.

Taken together, these results suggest that SCRIB binding restricts the conformational flexibility of PP1, particularly around its active site and phosphate-coordinating residues. Although the canonical geometry of the PP1 active site remains intact in the complex, the stabilization limits the conformational plasticity typically induced by ligand binding. This constraint affects subtle loop rearrangements and side-chain rotamer shifts, which are known to influence the dynamics of individual secondary structure elements (14).

### Insights on the architecture of the paradoxical MRAS, SHOC2, and SCRIB complex

SCRIB and SHOC2 exert opposing functions on ERK pathway activation, primarily through their opposing effects on PP1-mediated dephosphorylation of RAF family proteins. In the presence of GTP-bound MRAS, SHOC2 potentiates RAF family activation by facilitating PP1-dependent dephosphorylation of BRAF at Ser365 (and equivalent sites in its orthologues) (23, 24, 25, 26, 34). Conversely, SCRIB antagonizes this dephosphorylation process. Intriguingly, despite their opposing roles, SHOC2 and SCRIB have been shown to form a paradoxical complex in cells (21, 53). The molecular architecture of this complex remains unresolved.

Structural superimposition of the cryo-EM SHOC2–MRAS–PP1 holoenzyme (PDB 7UPI) with the SCRIB–PP1 complex reveals extensive overlap in their PP1-binding interfaces (26). Both SHOC2’s N-terminal V x F motif and SCRIB’s KGVSF motif engage the RVxF-binding pocket on PP1, albeit from opposite directions. Moreover, the LRR domains of SHOC2 and SCRIB, which are similar in size and curvature, bind overlapping regions on the PP1 surface *via* their concave faces (Fig. 2*C*). Additionally, the positioning of MRAS in the SHOC2 complex sterically clashes with the first six LRR repeats of SCRIB. Together, these structural constraints indicate that SHOC2–MRAS and SCRIB cannot simultaneously bind to the same PP1 molecule, establishing a model in which activation and inhibition of PP1 by these regulators are mutually exclusive.

### Human mutations in SCRIB map to its interface with PP1

Despite SCRIB’s well-established roles in cellular polarity and migration, and as a tumor suppressor in *Drosophila* and mouse models (31, 43, 54, 55, 56, 57, 58, 59, 60, 61, 62, 63), human SCRIB variants that affect its action on PP1 have not yet been reported. We identified 286 single-nucleotide variants in *SCRIB* listed in ClinVar. Of those variants, 264 are classified as variants of uncertain significance (VUS), and the remaining 22 variants are annotated as benign or likely benign.

Based on our cryo-EM structure, 11 ClinVar variants are located at the SCRIB–PP1 interface, whereas nine are positioned at the LRR–PDZ1 surface (Fig. S13 and Supplementary Data). All of these variants are currently classified as VUS. Within the SCRIB–PP1 complex, ten variants are situated on the concave LRR surface that faces PP1: I109V, H132Y, T155I, E229G, Q275R, S292F, R321Q, S338R, A392V, and Q393R. Among these, I109V, H132Y, T155I, A392V, and S292F are not predicted to directly impact PP1 binding. However, E229G, Q275R, and R321Q may impair binding or inhibition by altering the surface stereochemistry near the PP1 interface. S338R and Q393R may enhance PP1 association by replacing weak hydrogen bonds (to PP1 E44 and E54, respectively) with electrostatic interactions. Four LRR variants (V14M, Y34S, E30D, and I31V) occur at the interface with PDZ1. V14M and Y34S may destabilize PDZ1 association, as both interact with PDZ1 residue T749, which anchors the domain to the LRR. E30D and I31 V are predicted to destabilize the first LRR motif by disrupting intra-chain ionic (E30–K19) and hydrophobic (I31–hydrophobic core of the first two LRR motifs) interactions, potentially impairing PDZ1 binding indirectly.

Within PDZ1, R762W likely disrupts electrostatic contacts with LRR residue E40. Additionally, G747S, P750S, E756K, and F759L may locally alter the structure and dynamics of the PDZ1 loop region that interfaces with the LRR. Finally, R829Q is located in the C-terminal extension following PDZ1, where R829 engages in electrostatic interactions with PP1 residue D253. Substitution with glutamine may preserve hydrogen bonding but would eliminate the ionic interaction. We produced this variant in the background of the KGVSF–PDZ1+ peptide (residues 701–840). MST and ITC showed that the R829Q mutation destabilized binding to PP1 by a *ΔΔG* of approximately +3 kJ/mol (Fig. 2*A*, Table 1, and Fig. S4*B*). ITC further showed that this loss in affinity is driven by a pronounced enthalpic penalty (*ΔΔH* = +22.5 kJ/mol) that is largely counterbalanced by a favorable entropic gain (*ΔTΔS* = +19.1 kJ/mol). This thermodynamic profile exhibits a classic enthalpy-entropy compensation signature, which is consistent with the disruption of a solvent-exposed surface salt bridge, as predicted by our cryo-EM structure.

Taken together, this structural analysis suggests that a subset of SCRIB variants may alter its ability to associate with, and thereby inhibit, PP1, raising the possibility that some of these variants could contribute to pathogenic phenotypes.

## Conclusion

Our study reveals that SCRIB engages PP1 through a multipronged interaction involving its LRR domain, the PDZ1 domain, and the flanking N- and C-terminal extensions. The N-terminal extension includes the ^705^KGVSF sequence that functionally replaces the canonical RVxF motif, structurally corroborating the mutational analysis by Young *et al.* (2013) (21). The KGVSF motif and C-terminal extension are well supported by density and mutational analysis (*i.e.*, the KGVSA and R829Q mutations) and anchor the fragment with a high-confidence sequence register. The PDZ1 domain itself exhibits lower local resolution due to its flexible tethering. Consequently, the positioning of the main chain and side-chain rotamers within the central PDZ1 fold relies on high-scoring structural models guided by conservative adjustments into the residual cryo-EM density.

The apparent composite binding mode between SCRIB and PP1 aligns with and explains previous mutational analyses (21, 40). Individually, each of these SCRIB regions bound PP1 only weakly, supporting that a biologically functional interaction requires the concerted contribution of all three elements. This architecture suggests that perturbing a single interface could sufficiently destabilize the SCRIB–PP1 complex to modulate its biological function. This feature might be used for regulatory purposes. For instance, we observed that the spatial arrangement of SCRIB’s LRR and PDZ1 domains is incompatible with simultaneous binding of canonical ligands to PDZ1. Therefore, such ligands, alone or in synergy with other interactions, could prime SCRIB for release of PP1, and commit SCRIB to different cellular roles and loci. Such a mechanism would be compatible with the literature, as known PDZ1 ligands are not involved in the SHOC2/SCRIB/ERK regulation pathway (30, 39, 64).

The multipronged nature of SCRIB–PP1 binding also has implications for its competition with SHOC2. Structural overlap in their PP1-binding surfaces precludes simultaneous binding of both regulators to the same PP1 molecule, explaining their mutual exclusive control of PP1 (see Fig. 2*C*). Interestingly, previous mutational experiments showed that SCRIB residues 422 to 650 contain a SHOC2-binding region (21). Our cryo-EM structure and AlphaFold modelling suggest that this region forms a flexible linker between the SCRIB LRR and PDZ1 domains, not involved in PP1 binding. AlphaFold further predicts that SCRIB residues 614 to 634 form a loop–helix element that hydrophobically associates with the C-terminal helices that cap the SHOC2 LRR, while positively charged residues 604 to 613 may engage in electrostatic interactions with SHOC2’s convex LRR surface (ipTM = 0.8 for residues 604–634 binding SHOC2; Fig. S14, *A* and *B*). Based on these predictions, we propose a model in which, in the absence of GTP-bound MRAS, SCRIB sequesters and inactivates PP1 while using its LRR–PDZ1 linker to recruit SHOC2 (Fig. 5*E*). Upon activation, MRAS helps SHOC2 to displace SCRIB, allowing SHOC2 to activate PP1 and promote RAF dephosphorylation (Fig. 5*E*). The ability of SCRIB to trap PP1 in an active-like conformation may prime PP1 for fast activation upon handover to SHOC2–MRAS. While consistent with prior findings (21), this model remains to be validated experimentally, which lies beyond the scope of the present work.

The mode of inhibition used by SCRIB is mechanistically distinct from previously described PP1 inhibitors, which act by occluding the substrate-binding grooves or the active site, or by destabilizing the catalytic site and compromising the coordination of the catalytic ions (12, 22, 33, 35, 44, 45, 46). Instead, enzymatic and computational analyses show that SCRIB-mediated inhibition only marginally relies on reduced substrate binding. Rather, the dominant effect is a reduction in catalytic turnover. Based on our computational analyses, we propose that this reduction is caused by the conformational rigidification and modified electrostatics of the PP1 active site. These effects, which warrant experimental validation, would alter the environment of the catalytic ions and hinder the subtle structural adjustments normally associated with ligand binding (14). Our structural data thus expand the known repertoire of PP1 regulatory strategies, identifying SCRIB as a first inhibitor that maintains PP1 in an active-like, ion-loaded conformation while suppressing its catalytic output.

Our biochemical and structural findings provide a molecular context for several cellular roles of SCRIB. Previously, cellular studies demonstrated that SCRIB acts as a negative regulator of Raf/MEK/ERK signaling, where its loss or displacement correlates with hyperactivated ERK and oncogene-induced cell transformation (21, 32). By demonstrating that the SCRIB–PP1 complex achieves a twentyfold reduction in catalytic turnover (*k*_*cat*_) rather than preventing substrate recruitment, our data clarify how SCRIB can robustly dampen baseline ERK signaling in a cellular environment without completely occluding the capability of PP1 to interact with surrounding proteins.

Furthermore, the observation that SCRIB traps PP1 in an ion-loaded, 'active-like' state carries physiological significance for spatial cellular signaling. This priming likely facilitates rapid, localized activation spikes when upstream signals, such as GTP-bound MRAS, prompt SHOC2 to displace SCRIB at the plasma membrane (21). SCRIB is also involved in maintaining basolateral membrane networks and apicobasal polarity *via* its association with PP1 (40). Our kinetic data provide a structural mechanism behind the 'latent pool' hypothesis proposed by Troyanovsky *et al.* (2021) (40), wherein the basolateral cell cortex uses SCRIB to house a pool of fully metal-coordinated, but catalytically repressed PP1. By keeping PP1 in a primed state, SCRIB would assist the cell to transition rapidly from cell-polarity signaling to growth-factor-induced proliferation without the kinetic delay of re-loading catalytic metal ions into the phosphatase core.

The proposed capability of SCRIB to restrict the conformational dynamics of PP1, alongside its role in spatial scaffolding, parallels its capability to conformationally lock and guide the activity of the kinase YES1 (65). However, in contrast to its inhibitory effect on PP1, SCRIB stabilizes the catalytically active conformation of YES1. Intriguingly, SCRIB also anchors (but does not inhibit) the phosphatases PHLPP1 and PTEN at the cell membrane to repress AKT activation (66, 67). Consequently, mislocalized SCRIB, a common hallmark of many cancers, exerts its oncogenic effects by geographically isolating PHLPP1 and PTEN rather than through direct catalytic inhibition (43, 66, 67). Further investigations are required to decipher why SCRIB modulates the enzymatic activity of only a subset of its client proteins.

Finally, our structural insights provide a framework for interpreting pathogenic SCRIB variants catalogued in the ClinVar database. Clinical annotation of these variants is currently limited, preventing detailed genotype–phenotype correlations. Experimental assessment of mutations at the SCRIB–PP1 interface was also not feasible in our co-expression system, as disruption of PP1 binding resulted in protein insolubility. Nevertheless, analysis of the clinically observed R829Q mutation in the C-terminal extension of the PDZ1 domain demonstrated a measurable effect on PP1 binding *in vitro*. Although broader experimental validation will be required to ascertain pathological effects, our work offers a molecular framework for understanding how disease-associated SCRIB variants may perturb PP1 regulation and related cellular functions, thereby providing a basis for future experimental and clinical studies.

## Experimental procedures

### Expression and purification of SCRIB PDZ domain constructs

SCRIB PDZ1 comprising the N-terminal KGVSF motif (residues 701–820; referred to as KGVSF-PDZ1), the fragment comprising KGVSF-PDZ1 and the C-terminal tail (residues 700–840; named KGVSF-PDZ1+), the KGVSF-PDZ1+ fragment with the R829Q mutation (KGVSF-PDZ1+_R829Q_), and the SCRIB PDZ4 comprising the C-terminal KLDY motif (residues 1101–1280; PDZ4-KLDY) were ordered from Twist Bioscience, cloned into the pJEx411c vector with an N-terminal GST tag and a PreScission protease cleavage site. These constructs were expressed in *E. coli* BL21(DE3) cells.

Cells were cultured in LB broth supplemented with kanamycin at 37 °C while shaking at 200 RPM. Protein expression was induced with 250 μM isopropyl β-D-1-thiogalactopyranoside (IPTG) when the optical density at 600 nm (OD_600_) reached 0.6 to 0.8. After induction, cultures were incubated at 16 °C, shaking at 200 RPM for 16 h before harvesting. For purification, cell pellets were resuspended in purification buffer (50 mM Tris pH 8.0, 500 mM NaCl, 2 mM dithiothreitol (DTT)) supplemented with EDTA-free protease inhibitor cocktail [Roche], 0.2% Triton X-100, and benzonase nuclease [Merck Millipore]. Cells were lysed by sonication on ice for 10 min at 35% amplitude. The lysate was clarified by centrifugation, and the supernatant was applied to a GST-affinity column containing glutathione sepharose beads (Cytiva) for 1.5 h at 4 °C with gentle shaking. After discarding the flow-through, the column was washed and incubated overnight at 4 °C with 3C protease in 20 ml of purification buffer to cleave the GST tag. The eluate was concentrated using Amicon Ultra centrifugal filters (Merck Millipore), then further purified by SEC using a HiLoad 16/600 Superdex 200 pg column (GE Healthcare) equilibrated in SEC buffer (20 mM HEPES pH 7.5, 150 mM NaCl, 2 mM DTT). Protein purity was confirmed by SDS-PAGE. Fractions containing pure protein were pooled, concentrated, snap-frozen in liquid nitrogen, and stored at −80 °C. The recombinant KGVSF-PDZ1+_R829Q_ mutant protein was initially subjected to anion-exchange chromatography using a Mono Q 5/50 Gl column (Cytiva). As the protein did not separate into a sufficiently homogeneous peak, the pooled fractions were subsequently applied to a Mono S 5/50 Gl cation-exchange column (Cytiva). Under the selected buffer conditions, KGVSF-PDZ1+_R829Q_ was recovered in the flow-through and wash fractions, while contaminating proteins remained bound to the resin. The protein-containing fractions were pooled and concentrated using Amicon Ultra centrifugal filter units (Merck Millipore) before further purification by size-exclusion chromatography on a Superdex 200 Increase 10/300 Gl column (Cytiva) equilibrated with 20 mM HEPES (pH 7.5), 150 mM NaCl, and 2 mM DTT. The purity and homogeneity of the purified protein were assessed by SDS–PAGE prior to downstream biophysical analyses.

### Expression and purification of PP1

Full-length PP1 (residues 1–330) was ordered from Twist Bioscience and cloned into the pJEx411c vector with an N-terminal 8 × His-MBP-TEV tag. The construct was expressed in *E. coli* BL21(DE3) cells grown in Terrific Broth media supplemented with 0.04% glycerol and 1 mM MnCl_2_. Protein expression was induced with 250 μM IPTG when the optical density at 600 nm (OD_600_) reached 0.8 to 1.0. After induction, cultures were incubated at 17 °C with shaking at 200 RPM for 16 h before harvesting. Cells were collected and stored at −80 °C. Cell pellets were resuspended in lysis buffer (50 mM Tris pH 8.0, 500 mM NaCl, 0.5 mM MnCl_2_, 10 mM imidazole, 3 mM 2-mercaptoethanol [BME], 5% glycerol) supplemented with EDTA-free protease inhibitor cocktail, 0.2% Triton X-100, and benzonase nuclease. Cells were lysed using a high-pressure cell disruptor (CF1, Biosystems Ltd). The lysates were passed through a HisTrap column (Cytiva) and eluted using a gradient of purification buffer containing 500 mM imidazole. The eluted protein was further purified by SEC using a HiLoad 16/600 Superdex 200 pg column in the purification buffer. The clean fractions were pooled, and the 8×His-MBP-TEV tag was cleaved using TEV protease. The protein was then passed through a second HisTrap column (Cytiva) to remove the cleaved tag. Pure PP1 was concentrated and run again on a HiLoad 16/600 Superdex 200 pg column in SEC buffer (20 mM HEPES pH 7.5, 500 mM NaCl, 0.5 mM TCEP, 5% glycerol, 0.5 mM MnCl_2_). Protein purity was confirmed by SDS-PAGE. Fractions containing pure protein were pooled, concentrated, snap-frozen in liquid nitrogen, and stored at −80 °C.

### Expression and purification of SCRIB–PP1 complex

SCRIB (residues 13–1530) and PP1 (full-length) were cloned into the pFastBac Dual expression vector (10,359–016, Gibco) for use with the Bac-to-Bac baculovirus expression system. SCRIB was cloned under the p10 promoter with an N-terminal Strep tag, and PP1 was cloned under the polyhedrin (PH) promoter with a His tag. Recombinant bacmid was generated in *DH10Bac* cells (10,361,012, Gibco), and the complex was subsequently expressed in *Spodoptera frugiperda* 9 (Sf9) cells. Cells were harvested and stored at −80 °C. Cell pellets were resuspended in Strep purification buffer (50 mM Tris pH 8.0, 300 mM NaCl, 2 mM DTT) supplemented with EDTA-free protease inhibitor cocktail, benzonase nuclease, and 0.5 mM MnCl_2_. Cells were lysed by sonication on ice for 3 min at 25% amplitude. The lysate was clarified and passed through a StrepTrap XT column (Cytiva), and the protein complex was eluted using 50 mM biotin (VWR). SCRIB co-eluted with PP1 as a complex, which was further purified by size-exclusion chromatography (SEC) using Superose 6 (Cytiva) in SEC buffer (20 mM Tris pH 7.5, 150 mM NaCl, 2 mM DTT).

### Expression and purification of SCRIB_LRR

SCRIB LRR (residues 23–428; referred to as LRR) was cloned into the pFastBac Dual expression vector for the Bac-to-Bac baculovirus expression system. LRR was cloned under the p10 promoter with an N-terminal Strep tag. Recombinant bacmid was produced in *DH10Bac* cells, and the protein was expressed in Sf9 cells. Cells were harvested and stored at −80 °C. Cell pellets were resuspended in Strep purification buffer (50 mM Tris pH 8.0, 300 mM NaCl, 2 mM DTT), supplemented with EDTA-free protease inhibitor cocktail and benzonase nuclease. Lysis was performed by sonication on ice for 3 min at 25% amplitude. The lysate was clarified and passed through a StrepTrap XT column (Cytiva), and LRR was eluted using 50 mM biotin (VWR). Purified LRR was further cleaned by SEC using a HiLoad 16/600 Superdex 200 pg column in SEC buffer (20 mM Tris pH 7.5, 150 mM NaCl, 2 mM DTT).

### Synthetic peptides

Peptides were commercially synthesized by GenScript. Sequences used are: pBRAF peptide (^358^GQRDRSS{pSER}APNVH^370^), BRAF (^358^GQRDRSSSAPNVH^370^), KVGSF motif(^695^EGAVVSAPSVKGVSFDQANN^714^), KVGSA motif (^695^EGAVVSAPSVKGVSADQANN^714^), KLDY motif (^1255^EAAGRGLQPLKLDYRALAAVPSAG^1278^), SCRIB-derived sequence proposed by AlphaFold to bind to the PP1 active site (^469^GLQRRATPHPSELK^482^).

### Cryo-EM specimen preparation and data collection

Fresh SEC peak fractions of the SCRIB–PP1 complex were used for Cryo-EM specimen preparation. The complex was diluted to a concentration of 0.5 mg/ml in SCRIB–PP1 SEC buffer. Cryo-EM specimens were prepared at 4 °C under 100% humidity using a Vitrobot Mark IV (Thermo Fisher Scientific). A volume of 3 μl of the sample was applied to glow-discharged gold grids (Quantifoil R1.2/1.3, Au 200 mesh) and blotted for 3 s before being plunge-frozen in liquid ethane.

Data collection was performed using a Titan Krios G4 TEM operating at 300 kV, equipped with a Selectris X energy filter and a Falcon 4i direct electron detector. A total of 5020 movies were acquired at a magnification of 130k, corresponding to a pixel size of 0.93 Å/pixel at the specimen level. An electron flux of 4.5 e^-^/pixel/s resulted in a total dose of 36.4 e^-^/Å^2^ over a 7-s exposure. Data acquisition utilized aberration-free image shift (AFIS), with a nominal defocus range of −2.6 μm to −1.6 μm in 0.3 μm increments and an energy filter slit width of 10 eV.

### Cryo-EM image processing

A comprehensive overview of the data processing pipeline for the SCRIB–PP1 complex is provided in the Supplementary Materials. Initially, motion correction was performed in RELION (68) and the corrected micrographs were imported into cryoSPARC (v4.7) and picked using Blob Picker for preliminary 2D classification to generate templates (69). Subsequently, the full set of 5020 raw movies was transferred to cryoSPARC, where Patch Motion Correction and Patch CTF Estimation were applied using default parameters. Particles were picked using Template Picker and extracted using a box size of 256 pixels, followed by two rounds of 2D classification and *ab initio* reconstruction with four classes. Particles from classes 1 and 2 were subjected to homogeneous refinement to generate higher quality references and used for three rounds of heterogeneous refinement using an overpicked particle stack with four additional junk volumes to remove junk particles. The particles were then subjected to additional rounds of 2D classification to further improve homogeneity, followed by non-uniform refinement (70). A template set derived from the refined particles was used for improved particle selection through iterative *ab initio* reconstruction, 2D classification, non-uniform refinement, and local refinement. These curated particles were then used to train Topaz (71). Particles extracted using Topaz underwent 2D classification, *ab initio* reconstruction, and heterogeneous refinement. The best-resolved class was subjected to further 2D classification, heterogeneous refinement, and non-uniform refinement, yielding a stack of 118,595 high-quality particles. These particles were further refined using reference-based motion correction. A soft mask was generated in UCSF ChimeraX using the molmap command based on an AlphaFold-predicted model of the SCRIB–PP1 complex (72, 73). The mask was imported into cryoSPARC for non-uniform refinement without dynamic masking. Final particle curation included two additional rounds of 2D classification, yielding 100,498 particles. To visualize potential active site occlusion, 3D variability analysis (3DVA) was performed in cryoSPARC using a filter resolution of 4 Å and visualized using 3DVA Display in Simple mode (74). To remove noise, junk, and misaligned particles, high-resolution heterogeneous *ab initio* reconstruction (HR-HAIR) (75) was performed, with the best class yielding 43,433 particles. The resulting particle stack was further refined using local refinement, yielding the final map at 3.5 Å resolution. See Table S1. for cryo-EM data collection, refinement and validation statistics.

### Model building, fitting, and refinement

An initial atomic model of the SCRIB–PP1 complex was generated using the AlphaFold prediction server (73). The predicted structure was rigid-body docked into the cryo-EM density map using Phenix Dock in Map. This preliminary placement was further refined by docking into a map filtered for model building (EM-ready) (76) followed by manual model adjustment and rebuilding in Coot (77). The refined model was then re-docked into the final cryo-EM map, and Phenix Real Space Refinement was used to optimize the fit of the model to the density (78). Final structural validation was done in Phenix, and visualization was performed using UCSF ChimeraX and PyMOL (72, 78).

### Isothermal titration calorimetry (ITC)

Proteins were dialyzed overnight at 4 °C against degassed ITC buffer containing 20 mM HEPES (pH 7.5), 500 mM NaCl, 0.5 mM TCEP, 0.5 mM MnCl_2_, and 5% glycerol. ITC measurements were performed in triplicate using a MicroCal PEAQ-ITC instrument (Malvern Panalytical). In each experiment, 20 μM PP1 was loaded into the cell, while KGVSF-PDZ1, KGVSF-PDZ1+_R829Q_ were titrated from the syringe at concentrations of 400 μM (wild type) or 200 μM (KGVSF-PDZ1+_R829Q_), and PDZ4-KLDY was titrated from the syringe at concentration of 500 μM. For the KGVSF motif and KLDY motif, syringe concentrations were 750 μM and 500 μM, respectively, with PP1 maintained at 20 μM in the cell. All measurements were conducted at 25 °C with a stirring speed of 750 rpm. A total of 19 injections of 2 μl each were delivered at 150-s intervals. Data analysis and figure preparation were carried out using the MicroCal PEAQ-ITC Analysis Software (Malvern Panalytical).

### Microscale thermophoresis

Recombinant human PP1 and SCRIB:PP1 were fluorescently labeled using the Monolith Protein Labeling Kit RED-NHS second Generation (NanoTemper Technologies) according to the manufacturer's instructions. Labelled PP1 was diluted to a final concentration of 100 nM in MST buffer consisting of 20 mM HEPES (pH 7.5), 500 mM NaCl, and 0.5 mM TCEP, 0.5 mM MnCl_2_, and 5% glycerol. Unlabeled binding partners, including the SCRIB-derived KVGSF and KVGSA peptides, PDZ1, PDZ1+, and KGVSF-PDZ1+_R829Q_ proteins, were prepared as serial dilutions in the same buffer and mixed with labelled PP1. 5 mM MnCl^2+^ ions were also tested against PP1 in the same conditions, with protein predialyzed with 10 mM EGTA. Samples were loaded into premium-coated capillaries (NanoTemper Technologies, Germany) and analyzed using a Monolith NT.115 instrument with 20% LED power and medium MST power. Binding curves were analyzed using MO.Affinity Analysis (NanoTemper Technologies) by fitting the normalized fluorescence changes to a one-site binding model to determine equilibrium dissociation constants.

### Phosphatase assays

All phosphatase assays were performed in 20 mM Tris (pH 8.0), 150 mM NaCl, and 2 mM MnCl_2_. For the pNPP hydrolysis assay, the following conditions were tested: PP1 alone, the SCRIB–PP1 complex, PP1 preincubated with the SCRIB LRR, PP1 preincubated with KGVSF–PDZ1, and PP1 preincubated with both LRR and KGVSF–PDZ1. Protein concentrations are indicated in the corresponding figures. Reactions were initiated by adding 25 μM pNPP and incubated for 20 min, after which absorbance was measured at 405 nm. For peptide dephosphorylation, PP1 was assayed alone, preincubated with individual SCRIB fragments (KGVSF–PDZ1, LRR, or both), MBP-tagged PP1, or PP1 saturated with a synthetic SCRIB peptide (residues 469–482). Each sample was incubated with 100 μM of pBRAF peptide (^358^GQRDRSS{pSER}APNVH^370^) for 10 min. Reactions were terminated by adding 5 μl of malachite green solution to a final volume of 20 μl, followed by a 20 min incubation at room temperature for color development. Phosphate release was quantified using a standard curve (0–160 μM inorganic phosphate), and absorbance was measured at 620 nm.

To assess the effect of SCRIB on PP1 catalytic efficiency, Michaelis–Menten kinetics were performed using pNPP as a substrate. Absorbance at 405 nm was recorded at 1 min intervals over 30 min. Turnover was calculated using the extinction coefficient for pNPP (18,000 M^−1^ cm^−1^ at 405 nm). Two independent experiments were carried out for each condition (PP1 and SCRIB–PP1). Kinetic parameters were derived, and plots were generated in GraphPad Prism 10.

### Inductively coupled plasma mass spectrometry

The manganese content of purified PP1 and SCRIB–PP1 proteins was determined by inductively coupled plasma mass spectrometry (ICP-MS) (48). Protein samples were prepared in a metal-depleted buffer (20 mM HEPES pH 7.5, 150 mM NaCl, 0.5 mM TCEP). Manganese concentration in the SEC-purified fractions was quantified using an ICP-MS instrument. Samples were diluted 100-fold into 2% (v/v) HNO_3_ (trace-metal grade) prior to analysis. A two-point external calibration curve was constructed using Mn^2+^ standard solutions (at 50 μM and 100 μM Mn^2+^) prepared by serial dilution of Mn^2+^. The calibration achieved a linear response over the measured range (R^2^ > 0.999), and calibration recovery was confirmed as 91.0% of the theoretical value, which is within the accepted ±15% tolerance for ICP-MS analysis. All values were corrected for this recovery factor. A procedural blank prepared in identical buffer conditions (protein-free buffer sample processed identically to protein samples) was measured alongside each batch to account for background Mn^2+^contamination from buffer components; the background contribution was subtracted from all sample values before stoichiometry calculation.

Proteinp concentrations were determined by UV absorbance at 280 nm (A280) using theoretical extinction coefficients calculated from the amino acid sequence. The molar stoichiometry of Mn2+ per protein molecule was calculated as.

where all concentrations are in μM. Reported values represent single measurements. Error bars represent propagated uncertainty combining the ICP-MS calibration uncertainty (±3%), the background subtraction uncertainty (±5% on the blank measurement), and the protein concentration uncertainty (±7% from A280), summed in quadrature.

### EPR spectroscopy and Mn^2+^ stoichiometry

Protein samples were prepared at the following concentrations: PP1 (27.5 μM), the SCRIB–PP1 SP (7.5 μM), and lysozyme as a non–Mn^2+^-binding control (30 μM). The lysozyme sample was incubated with 100 μM MnCl_2_in EPR buffer (20 mM HEPES pH 7.5, 150 mM NaCl, 0.5 mM TCEP) for 30 min on ice. To remove unbound and non-specifically associated Mn^2+^, all samples were dialysed overnight at 4 °C against 1 L Mn^2+^-free EPR buffer with two buffer exchanges. The dialyzed lysozyme sample served as the negative control for background Mn^2+^retention. Continuous-wave X-band EPR spectra were recorded in triplicate at room temperature on a Bruker EMX spectrometer operating at ∼9.4 GHz using the following parameters: sweep width 3000 G (2000–5000 G), modulation amplitude 14 G, microwave power 1 mW, and three accumulated scans. Spectra were acquired and processed using Bruker Xepr software. Quantification was performed using the sextet amplitude method, in which the peak-to-trough intensity within the 3300 to 3800 G region was extracted for each sample (79, 80). A calibration curve generated from four MnCl_2_ standards (20, 50, 100, and 500 μM) yielded a linear response (R^2^ = 0.99997) with a sensitivity of 1.07 × 10^−3^ EPR units per μM Mn^2+^. Sample Mn^2+^ concentrations were determined by interpolation against the average amplitude of two independent 100 μM Mn^2+^ standards, which agreed within 1.4%. Background Mn^2+^ (10.1 μM, determined from dialyzed lysozyme sample) was subtracted from all protein samples to obtain net protein-bound Mn^2+^. Stoichiometry was calculated by dividing the background-corrected Mn^2+^ concentration by the protein concentration determined by A_280_ absorbance prior to EPR analysis. Spectra were visualised in GraphPad Prism 10, and no baseline correction or spectral manipulation was applied to the data used for quantification.

### Protein structure preparation for molecular dynamics (MD) simulations

Four distinct systems of PP1 were prepared for molecular dynamics (MD) simulations (1): unbound PP1 (apo form) (2), PP1 in complex with the LRR and PDZ1 domains of SCRIB, and (3) PP1 bound to a phosphoserine-containing peptide, and (4) PP1-SCRIB complex bound to the same phosphoserine-containing peptide. The unbound PP1 system was based on the crystal structure with PDB: 6OBS (22). This structure contains a Y134A mutation, which was back-mutated to tyrosine using PyMOL to restore the wild-type sequence. The side chain orientation of the tyrosine was modeled to reflect the catalytically active conformation of PP1. Two manganese (Mn^2+^) ions and two structural water molecules, essential for enzymatic activity, were retained to maintain a biologically relevant configuration. For the PP1–SCRIB complex, the cryo-EM structure resolved with the LRR and PDZ1 domains was used. As the cryo-EM reconstruction lacked water molecules, two catalytic waters were manually placed by transferring coordinates from the PP1 crystal structure. The third and fourth systems, bound to the phosphoserine-containing peptide, were modeled using AlphaFold3 (73), which incorporated the peptide in an active-site-bound conformation. This construct mimicked substrate engagement at the catalytic site. As in the other systems, two Mn^2+^ ions and two structural water molecules were added based on conserved coordination in the active state of PP1.

### Metal center parameterization

Parameters for the binuclear manganese (Mn^2+^) active site of PP1, coordinated by two water molecules and six amino acid residues (Asp64, His66, Asp92, Asn124, His173, and His248) were generated using the Metal Center Parameter Builder (MCPB.py) module of the AMBER suite (81). To accurately capture the local coordination environment, a model was constructed including both Mn ions, two water ligands, and truncated/capped side chains of coordinating residues. Quantum mechanical (QM) calculations were performed in Gaussian16 (82) at the B3LYP/def2-SVP (83) level of theory. Solvation effects were modeled using the SMD implicit solvent model (with water) (84), and dispersion interactions were accounted for *via* the Grimme D3 correction (85). An ultrafine integration grid was employed. Geometry optimization was followed by vibrational frequency analysis to confirm energy minima and derive bonded parameters (*e.g.*, equilibrium bond lengths, angles, and force constants). Partial atomic charges were obtained by fitting the electrostatic potential using the Merz–Kollman scheme and the two-stage RESP fitting procedure implemented in MCPB.py. Following parameter derivation, an initial energy minimization revealed artifacts due to a bridging water molecule between Mn ions, resulting from 1 to 4 nonbonded interaction conflicts. To resolve this, a hybrid bonded/non-bonded approach was adopted. The two water molecules were modeled as non-bonded with harmonic positional restraints, using force constants derived from the bonded model to preserve proper coordination geometry.

### Molecular dynamics simulations

MD simulations were performed for the four systems: PP1 (apo), PP1-SCRIB complex, phosphopeptide-bound to PP1 and phosphopeptide-bound to PP1-SCRIB complex. Each system was simulated in four independent replicas for 1000 ns (1 μs each), using a 2 fs timestep. Simulations were performed using GROMACS 2024.2 (86), with the Amber19SB force field (87) for proteins and TIP3P water model (88). Parameters for the binuclear Mn^2+^ center and coordinating waters were generated as described above using MCPB.py (81). Systems were solvated in periodic boxes and neutralized by adding Na^+^ and Cl^-^ counterions. Electrostatics were treated using the PME algorithm (cutoff: 0.9 nm), with a reciprocal grid of 64 × 60 × 60 and fourth-order B-spline interpolation (89). van der Waals interactions used a 0.93 nm cutoff. All hydrogen-containing bonds were constrained *via* the LINCS algorithm (90). Temperature was maintained at 310 K with the V-rescale thermostat (91), and pressure at 1 atm using the C-rescale barostat (92), under periodic boundary conditions. Each system underwent steepest descent energy minimization followed by multi-step equilibration. Snapshots were saved every 10 ps, and only the final 500 ns of each replica were used for analysis, yielding an equilibrated 2 μs trajectory per system.

As noted in the parameterization section, the coordinated water molecules were modeled using a restrained nonbonded approach. Harmonic restraints were applied at calculated equilibrium bond lengths and force constants using PLUMED v2.9 *via* the DISTANCE utility (93).

### Continuum electrostatic and SASA calculations

Continuum Electrostatic calculations have been performed using the NextGenPB solver (94). Analyzed molecular dynamics trajectory frames were stripped from explicit water molecules, then the corresponding.pqr file, containing atomic partial charges and radii consistent with the system, were passed to the code, which solves the Poisson-Boltzmann equation. The ionic strength was assumed to be 145 mM of NaCl and the probe radius was set to 1.4 Å. The grid spacing of the solving regular Cartesian grid around the molecule was 0.5 Å, and then the homogeneous Dirichlet boundary conditions were applied on a large derefined grid as described in the main publication.

SASA calculations have been performed with the NanoShaper molecular surface building and analyzing tool, providing as input the same.pqr files used for electrostatic calculations (95).

### Variant retrieval and annotation

Variants in the human *SCRIB* gene (Scribble, HGNC: 30375) were retrieved from the ClinVar database (accessed August 11, 2025) and restricted to *Homo sapiens* single-nucleotide variants (SNVs). For each entry, we extracted the ClinVar Variation ID, dbSNP ID, GRCh38 and GRCh37 genomic coordinates, reference and alternate alleles, predicted molecular consequence, clinical significance, review status, and last-evaluated date. Variants flagged as somatic, multi-nucleotide substitutions, insertions or deletions, structural variants, or entries not mapping to *SCRIB* were excluded.

## Data availability

Cryo-EM reconstruction and atomic model have been deposited in the Electron Microscopy Data Bank (EMDB) and Protein Data Bank (PDB), respectively, under the following accession numbers: EMD-66672 and PDB 9X9Y9WMX. The MD simulation datasets that support the conclusions of this article are available in the Zenodo repository with the following https://doi.org/10.5281/zenodo.17277557.

## Supporting information

This article contains supporting information (21).

## Declaration of generative AI and AI-assisted technologies in the writing process

During the preparation of this work, the authors used Microsoft Copilot in order to improve the readability and language of the manuscript. AI produced text was reviewed and edited where necessary.

## Conflict of interest

The authors declare that they have no conflicts of interest with the contents of this article.

## Supporting information

Supplementary_revision

## References

[1] Hunter T. (2000). Signaling—2000 and beyond. Cell.

[2] Manning G., Whyte D.B., Martinez R., Hunter T., Sudarsanam S. (2002). The protein kinase complement of the human genome. Science.

[3] Ramazi S., Zahiri J. (2021). Post-translational modifications in proteins: resources, tools and prediction methods. Database J. Biol. Databases Curation.

[4] Pang K., Wang W., Qin J., Shi Z., Hao L., Hao Y. (2022). Role of protein phosphorylation in cell signaling, disease, and the intervention therapy. MedComm.

[5] Wilson L., Linley A., Hammond D., Hood F., Coulson J., MacEwan D. (2018). New perspectives, opportunities, and challenges in exploring the human protein kinome. Cancer Res..

[6] Chen M.J., Dixon J.E., Manning G. (2017). Genomics and evolution of protein phosphatases. Sci. Signal..

[7] Brautigan D.L., Shenolikar S. (2018). Protein Serine/Threonine phosphatases: keys to unlocking regulators and substrates. Annu. Rev. Biochem..

[8] Ferreira M., Beullens M., Bollen M., Van Eynde A. (2019). Functions and therapeutic potential of protein phosphatase 1: insights from mouse genetics. Biochim. Biophys. Acta Mol. Cell Res..

[9] Bollen M., Peti W., Ragusa M.J., Beullens M. (2010). The extended PP1 toolkit: designed to create specificity. Trends Biochem. Sci..

[10] Heroes E., Lesage B., Görnemann J., Beullens M., Van Meervelt L., Bollen M. (2013). The PP1 binding code: a molecular-lego strategy that governs specificity. FEBS J..

[11] Choy M.S., Hieke M., Kumar G.S., Lewis G.R., Gonzalez-DeWhitt K.R., Kessler R.P. (2014). Understanding the antagonism of retinoblastoma protein dephosphorylation by PNUTS provides insights into the PP1 regulatory code. Proc. Natl. Acad. Sci. U. S. A..

[12] Egloff M.P., Johnson D.F., Moorhead G., Cohen P.T., Cohen P., Barford D. (1997). Structural basis for the recognition of regulatory subunits by the catalytic subunit of protein phosphatase 1. EMBO J..

[13] Hendrickx A., Beullens M., Ceulemans H., Den Abt T., Van Eynde A., Nicolaescu E. (2009). Docking motif-guided mapping of the interactome of protein Phosphatase-1. Chem. Biol..

[14] Peti W., Nairn A.C., Page R. (2013). Structural basis for protein phosphatase 1 regulation and specificity. FEBS J..

[15] Peti W., Padi S.K.R., Page R. (2025). Combining cryo-electron microscopy (cryo-EM) with orthogonal solution state methods to define the molecular basis of the phosphoprotein phosphatase family regulation and substrate specificity. Curr. Opin. Struct. Biol..

[16] O’Connell N., Nichols S.R., Heroes E., Beullens M., Bollen M., Peti W., Page R. (2012). The molecular basis for substrate specificity of the nuclear NIPP1:PP1 holoenzyme. Struct. Lond. Engl..

[17] Scheinost L., Köhn M. (2025). The fascinating intricacy of pSer/Thr-Specific phosphatases and their higher-order complexes: emerging concepts. Biochemistry.

[18] Choy M.S., Srivastava G., Robinson L.C., Tatchell K., Page R., Peti W. (2024). The SDS22:PP1:I3 complex: SDS22 binding to PP1 loosens the active site metal to prime metal exchange. J. Biol. Chem..

[19] Bertran M.T., Mouilleron S., Zhou Y., Bajaj R., Uliana F., Kumar G.S. (2019). ASPP proteins discriminate between PP1 catalytic subunits through their SH3 domain and the PP1 C-tail. Nat. Commun..

[20] Rodriguez-Viciana P., Oses-Prieto J., Burlingame A., Fried M., McCormick F. (2006). A phosphatase holoenzyme comprised of Shoc2/Sur8 and the catalytic subunit of PP1 functions as an M-Ras effector to modulate raf activity. Mol. Cell.

[21] Young L.C., Hartig N., Muñoz-Alegre M., Oses-Prieto J.A., Durdu S., Bender S. (2013). An MRAS, SHOC2, and SCRIB complex coordinates ERK pathway activation with polarity and tumorigenic growth. Mol. Cell.

[22] Choy M.S., Moon T.M., Ravindran R., Bray J.A., Robinson L.C., Archuleta T.L. (2019). SDS22 selectively recognizes and traps metal-deficient inactive PP1. Proc. Natl. Acad. Sci. U. S. A..

[23] Liau N.P.D., Johnson M.C., Izadi S., Gerosa L., Hammel M., Bruning J.M. (2022). Structural basis for SHOC2 modulation of RAS signalling. Nature.

[24] Bonsor D.A., Alexander P., Snead K., Hartig N., Drew M., Messing S. (2022). Structure of the SHOC2–MRAS–PP1C complex provides insights into RAF activation and Noonan syndrome. Nat. Struct. Mol. Biol..

[25] Hauseman Z.J., Fodor M., Dhembi A., Viscomi J., Egli D., Bleu M. (2022). Structure of the MRAS–SHOC2–PP1C phosphatase complex. Nature.

[26] Kwon J.J., Hajian B., Bian Y., Young L.C., Amor A.J., Fuller J.R. (2022). Structure–function analysis of the SHOC2–MRAS–PP1C holophosphatase complex. Nature.

[27] Jones G.G., Del Río I.B., Sari S., Sekerim A., Young L.C., Hartig N. (2019). SHOC2 phosphatase-dependent RAF dimerization mediates resistance to MEK inhibition in RAS-mutant cancers. Nat. Commun..

[28] Lee S., Rauch J., Kolch W. (2020). Targeting MAPK signaling in cancer: mechanisms of drug resistance and sensitivity. Int. J. Mol. Sci..

[29] Pires H.R., Boxem M. (2018). Mapping the polarity interactome. J. Mol. Biol..

[30] Bonello T.T., Peifer M. (2019). Scribble: a master scaffold in polarity, adhesion, synaptogenesis, and proliferation. J. Cell Biol..

[31] Stephens R., Lim K., Portela M., Kvansakul M., Humbert P.O., Richardson H.E. (2018). The scribble cell polarity module in the regulation of cell signaling in tissue development and tumorigenesis. J. Mol. Biol..

[32] Nagasaka K., Seiki T., Yamashita A., Massimi P., Subbaiah V.K., Thomas M. (2013). A novel interaction between hScrib and PP1γ downregulates ERK signaling and suppresses oncogene-induced cell transformation. PLoS One.

[33] Goldberg J., Huang H.B., Kwon Y.G., Greengard P., Nairn A.C., Kuriyan J. (1995). Three-dimensional structure of the catalytic subunit of protein serine/threonine phosphatase-1. Nature.

[34] Snead K., Wall V., Ambrose H., Esposito D., Drew M. (2022). Polycistronic baculovirus expression of SUGT1 enables high-yield production of recombinant leucine-rich repeat proteins and protein complexes. Protein Expr. Purif..

[35] Ragusa M.J., Dancheck B., Critton D.A., Nairn A.C., Page R., Peti W. (2010). Spinophilin directs protein phosphatase 1 specificity by blocking substrate binding sites. Nat. Struct. Mol. Biol..

[36] Terrak M., Kerff F., Langsetmo K., Tao T., Dominguez R. (2004). Structural basis of protein phosphatase 1 regulation. Nature.

[37] Kumar G.S., Choy M.S., Koveal D.M., Lorinsky M.K., Lyons S.P., Kettenbach A.N. (2018). Identification of the substrate recruitment mechanism of the muscle glycogen protein phosphatase 1 holoenzyme. Sci. Adv..

[38] Lim K.Y.B., Gödde N.J., Humbert P.O., Kvansakul M. (2017). Structural basis for the differential interaction of scribble PDZ domains with the guanine nucleotide exchange factor β-PIX. J. Biol. Chem..

[39] How J.Y., Caria S., Humbert P.O., Kvansakul M. (2019). Crystal structure of the human scribble PDZ1 domain bound to the PDZ-binding motif of APC. FEBS Lett..

[40] Troyanovsky R.B., Indra I., Kato R., Mitchell B.J., Troyanovsky S.M. (2021). Basolateral protein scribble binds phosphatase PP1 to establish a signaling network maintaining apicobasal polarity. J. Biol. Chem..

[41] Fedoryshchak R.O., Přechová M., Butler A.M., Lee R., O'Reilly N., Flynn H.R. (2020). Molecular basis for substrate specificity of the Phactr1/PP1 phosphatase holoenzyme. eLife.

[42] Egloff M.P., Cohen P.T., Reinemer P., Barford D. (1995). Crystal structure of the catalytic subunit of human protein phosphatase 1 and its complex with tungstate. J. Mol. Biol..

[43] Santoni M.-J., Kashyap R., Camoin L., Borg J.-P. (2020). The scribble family in cancer: twentieth anniversary. Oncogene.

[44] Srivastava G., Choy M.S., Bolik-Coulon N., Page R., Peti W. (2023). Inhibitor-3 inhibits protein phosphatase 1 via a metal binding dynamic protein–protein interaction. Nat. Commun..

[45] Kita A., Matsunaga S., Takai A., Kataiwa H., Wakimoto T., Fusetani N. (2002). Crystal structure of the complex between Calyculin A and the catalytic subunit of protein phosphatase 1. Structure.

[46] Kelker M.S., Page R., Peti W. (2009). Crystal structures of protein Phosphatase-1 bound to Nodularin-R and tautomycin: a novel scaffold for structure-based drug design of Serine/Threonine phosphatase inhibitors. J. Mol. Biol..

[47] Dancheck B., Ragusa M.J., Allaire M., Nairn A.C., Page R., Peti W. (2011). Molecular investigations of the structure and function of the protein phosphatase 1−Spinophilin−Inhibitor 2 heterotrimeric complex. Biochemistry.

[48] Lane A., Gokhale A., Werner E., Roberts A., Freeman A., Robert B. (2022). Sulfur- and phosphorus-standardized metal quantification of biological specimens using inductively coupled plasma mass spectrometry. STAR Protoc..

[49] Hurley T.D., Yang J., Zhang L., Goodwin K.D., Zou Q., Cortese M. (2007). Structural basis for regulation of protein phosphatase 1 by Inhibitor-2. J. Biol. Chem..

[50] Foltran B.B., Gaspar J.P., Silva I.R.M., Pires H.M., Andrade F.B., Costa G.M. (2024). New insights into the putative role of leucine-rich repeat proteins of Leptospira interrogans and their participation in host cell invasion: an in silico analysis. Front. Cell. Infect. Microbiol..

[51] Miras I., Saul F., Nowakowski M., Weber P., Haouz A., Shepard W., Picardeau M. (2015). Structural characterization of a novel subfamily of leucine-rich repeat proteins from the human pathogen *Leptospira interrogans*. Acta Crystallogr. D Biol. Crystallogr..

[52] Santoni M.-J., Pontarotti P., Birnbaum D., Borg J.-P. (2002). The LAP family: a phylogenetic point of view. Trends Genet..

[53] Young L.C., Hartig N., Boned Del Río I., Sari S., Ringham-Terry B., Wainwright J.R. (2018). SHOC2–MRAS–PP1 complex positively regulates RAF activity and contributes to Noonan syndrome pathogenesis. Proc. Natl. Acad. Sci. U. S. A..

[54] Boëda B., Michel V., Etournay R., England P., Rigaud S., Mary H. (2023). SCRIB controls apical contractility during epithelial differentiation. J. Cell Biol..

[55] Shen H., Huang C., Wu J., Li J., Hu T., Wang Z. (2021). SCRIB promotes proliferation and metastasis by targeting Hippo/YAP signalling in colorectal cancer. Front. Cell Dev. Biol..

[56] Choi J., Troyanovsky R.B., Indra I., Mitchell B.J., Troyanovsky S.M. (2019). Scribble, Erbin, and Lano redundantly regulate epithelial polarity and apical adhesion complex. J. Cell Biol..

[57] Yates L.L., Schnatwinkel C., Hazelwood L., Chessum L., Paudyal A., Hilton H. (2013). Scribble is required for normal epithelial cell–cell contacts and lumen morphogenesis in the mammalian lung. Dev. Biol..

[58] Robinson A., Escuin S., Doudney K., Vekemans M., Stevenson R.E., Greene N.D.E. (2012). Mutations in the planar cell polarity genes CELSR1 and SCRIB are associated with the severe neural tube defect, craniorachischisis. Hum. Mutat..

[59] Pearson H.B., Perez-Mancera P.A., Dow L.E., Ryan A., Tennstedt P., Bogani D. (2011). SCRIB expression is deregulated in human prostate cancer, and its deficiency in mice promotes prostate neoplasia. J. Clin. Invest..

[60] Nola S., Sebbagh M., Marchetto S., Osmani N., Nourry C., Audebert S. (2008). Scrib regulates PAK activity during the cell migration process. Hum. Mol. Genet..

[61] Dow L.E., Elsum I.A., King C.L., Kinross K.M., Richardson H.E., Humbert P.O. (2008). Loss of human scribble cooperates with H-Ras to promote cell invasion through deregulation of MAPK signalling. Oncogene.

[62] Bilder D. (2004). Epithelial polarity and proliferation control: links from the Drosophila neoplastic tumor suppressors. Genes Dev..

[63] Murdoch J.N., Henderson D.J., Doudney K., Gaston-Massuet C., Phillips H.M., Paternotte C. (2003). Disruption of scribble (Scrb1) causes severe neural tube defects in the circletail mouse. Hum. Mol. Genet..

[64] Thüring E.-M., Hartmann C., Maddumage J.C., Javorsky A., Michels B.E., Gerke V. (2023). Membrane recruitment of the polarity protein scribble by the cell adhesion receptor TMIGD1. Commun. Biol..

[65] Zhao D., Yin Z., Soellner M.B., Martin B.R. (2021). Scribble sub-cellular localization modulates recruitment of YES1 to regulate YAP1 phosphorylation. Cell Chem. Biol..

[66] Feigin M.E., Akshinthala S.D., Araki K., Rosenberg A.Z., Muthuswamy L.B., Martin B. (2014). Mislocalization of the cell polarity protein scribble promotes mammary tumorigenesis and is associated with basal breast cancer. Cancer Res..

[67] Li X., Yang H., Liu J., Schmidt M.D., Gao T. (2011). Scribble-mediated membrane targeting of PHLPP1 is required for its negative regulation of Akt. EMBO Rep..

[68] Kimanius D., Dong L., Sharov G., Nakane T., Scheres S.H.W. (2021). New tools for automated cryo-EM single-particle analysis in RELION-4.0. Biochem. J..

[69] Punjani A., Rubinstein J.L., Fleet D.J., Brubaker M.A. (2017). cryoSPARC: algorithms for rapid unsupervised cryo-EM structure determination. Nat. Methods.

[70] Punjani A., Zhang H., Fleet D.J. (2020). Non-uniform refinement: adaptive regularization improves single-particle cryo-EM reconstruction. Nat. Methods.

[71] Bepler T., Kelley K., Noble A.J., Berger B. (2020). Topaz-Denoise: general deep denoising models for cryoEM and cryoET. Nat. Commun..

[72] Pettersen E.F., Goddard T.D., Huang C.C., Meng E.C., Couch G.S., Croll T.I. (2021). UCSF ChimeraX: structure visualization for researchers, educators, and developers. Protein Sci. Publ. Protein Soc..

[73] Abramson J., Adler J., Dunger J., Evans R., Green T., Pritzel A. (2024). Accurate structure prediction of biomolecular interactions with AlphaFold 3. Nature.

[74] Punjani A., Fleet D.J. (2021). 3D variability analysis: resolving continuous flexibility and discrete heterogeneity from single particle cryo-EM. J. Struct. Biol..

[75] Kim K., Li H., Clarke O.B. (2025). High-resolution ab initio reconstruction enables cryo-EM structure determination of small particles. bioRxiv.

[76] He J., Li T., Huang S.-Y. (2023). Improvement of cryo-EM maps by simultaneous local and non-local deep learning. Nat. Commun..

[77] Emsley P., Cowtan K. (2004). Coot: model-building tools for molecular graphics. Acta Crystallogr. D Biol. Crystallogr..

[78] Liebschner D., Afonine P.V., Baker M.L., Bunkóczi G., Chen V.B., Croll T.I. (2019). Macromolecular structure determination using X-rays, neutrons and electrons: recent developments in Phenix. Acta Crystallogr. Sect. Struct. Biol..

[79] Reed G.H., Markham G.D., Berliner L.J., Reuben J. (1984). Biological Magnetic Resonance: Volume 6.

[80] Wuebben C., Schiemann O., Steger G., Rosenbach H., Span I. (2022). Dnazymes: Methods and Protocols.

[81] Li P., Merz K.M. (2016). MCPB.py: a python based metal center parameter builder. J. Chem. Inf. Model..

[82] Frisch M.J., Trucks G.W., Schlegel HB., Scuseria G.E., Robb M., Cheeseman J.R. (2016). Gaussian 16 Rev. C.01.

[83] Weigend F., Ahlrichs R. (2005). Balanced basis sets of split valence, triple zeta valence and quadruple zeta valence quality for H to Rn: design and assessment of accuracy. Phys. Chem. Chem. Phys..

[84] Marenich A.V., Cramer C.J., Truhlar D.G. (2009). Universal solvation model based on solute electron density and on a continuum model of the solvent defined by the bulk dielectric constant and atomic surface tensions. J. Phys. Chem. B.

[85] Grimme S., Antony J., Ehrlich S., Krieg H. (2010). A consistent and accurate ab initio parametrization of density functional dispersion correction (DFT-D) for the 94 elements H-Pu. J. Chem. Phys..

[86] Abraham M.J. (2015). GROMACS: high performance molecular simulations through multi-level parallelism from laptops to supercomputers. SoftwareX.

[87] Tian C., Kasavajhala K., Belfon K.A.A., Raguette L., Huang H., Migues A.N. (2020). ff19SB: amino-acid-specific protein backbone parameters trained against quantum mechanics Energy surfaces in solution. J. Chem. Theor. Comput..

[88] Jorgensen W.L., Chandrasekhar J., Madura J.D., Impey R.W., Klein M.L. (1983). Comparison of simple potential functions for simulating liquid water. J. Chem. Phys..

[89] Darden T., York D., Pedersen L. (1993). Particle mesh Ewald: an *N* ⋅log(*N*) method for Ewald sums in large systems. J. Chem. Phys..

[90] Hess B., Bekker H., Berendsen H.J.C., Fraaije J.G.E.M. (1997). LINCS: a linear constraint solver for molecular simulations. J. Comput. Chem..

[91] Bussi G., Donadio D., Parrinello M. (2007). Canonical sampling through velocity rescaling. J. Chem. Phys..

[92] Bernetti M., Bussi G. (2020). Pressure control using stochastic cell rescaling. J. Chem. Phys..

[93] Tribello G.A., Bonomi M., Branduardi D., Camilloni C., Bussi G. (2014). Plumed 2: new feathers for an old bird. Comput. Phys. Commun..

[94] Di Florio V., Ansalone P., Siryk S.V., Decherchi S., Falco C.D., Rocchia W. (2025). NextGenPB: an analytically-enabled super resolution tool for solving the poisson-Boltzmann equation featuring local (de)refinement. Comput. Phys. Commun..

[95] Decherchi S., Rocchia W. (2013). A general and robust ray-casting-based algorithm for triangulating surfaces at the nanoscale. PLoS One.

