# Supplementary material for: SCRIB inhibits PP1 phosphatase activity without blocking or structurally altering its active site
Source: J Biol Chem. 2026 Sep 23;302(11):113599. doi: 10.1016/j.jbc.2026.113599 (PMC13737739; doi:10.1016/j.jbc.2026.113599)
Supplement: Supplementary file 1 [file mmc1.pdf]

Supplementary Information

SCRIB inhibits PP1 phosphatase activity without blocking or structurally altering its active site

AUTHORS: Siba Alharbi[1,$], Amit Kumawat[2,#], Brandon Huntington[1,#], Afaque A. Momin[1,#], Shaden Alfaiyz[1], Vincenzo di Florio[3,5], Marta Rigoli[3], Kelly Cardona Londono[1], Walter Rocchia[3], Anandsukeerthi Sandholu[1,$,], Stefan T. Arold[1,*]*

AFFILIATIONS:

[1] KAUST Center of Excellence for Smart Health, Biological and Environmental Science and Engineering Division, King Abdullah University of Science and Technology (KAUST), Thuwal 23955-6900, Kingdom of Saudi Arabia.

[2] Department of Physics, University of Cagliari, Cagliari, Italy

[3] CONCEPT Lab, Istituto Italiano di Tecnologia, 16152 - Genova, Italy

[4] MOX - Dept. of Mathematics, Politecnico di Milano, 20133 - Milan, Italy

List of contents:

Supplementary Figure 1
Supplementary Figure 2
Supplementary Figure 3
Supplementary Figure 4
Supplementary Figure 5
Supplementary Figure 6
Supplementary Figure 7
Supplementary Figure 8
Supplementary Figure 9
Supplementary Figure 10
Supplementary Figure 11
Supplementary Figure 12
Supplementary Figure 13
Supplementary Figure 14
Supplementary Table 1
Supplementary Table 2

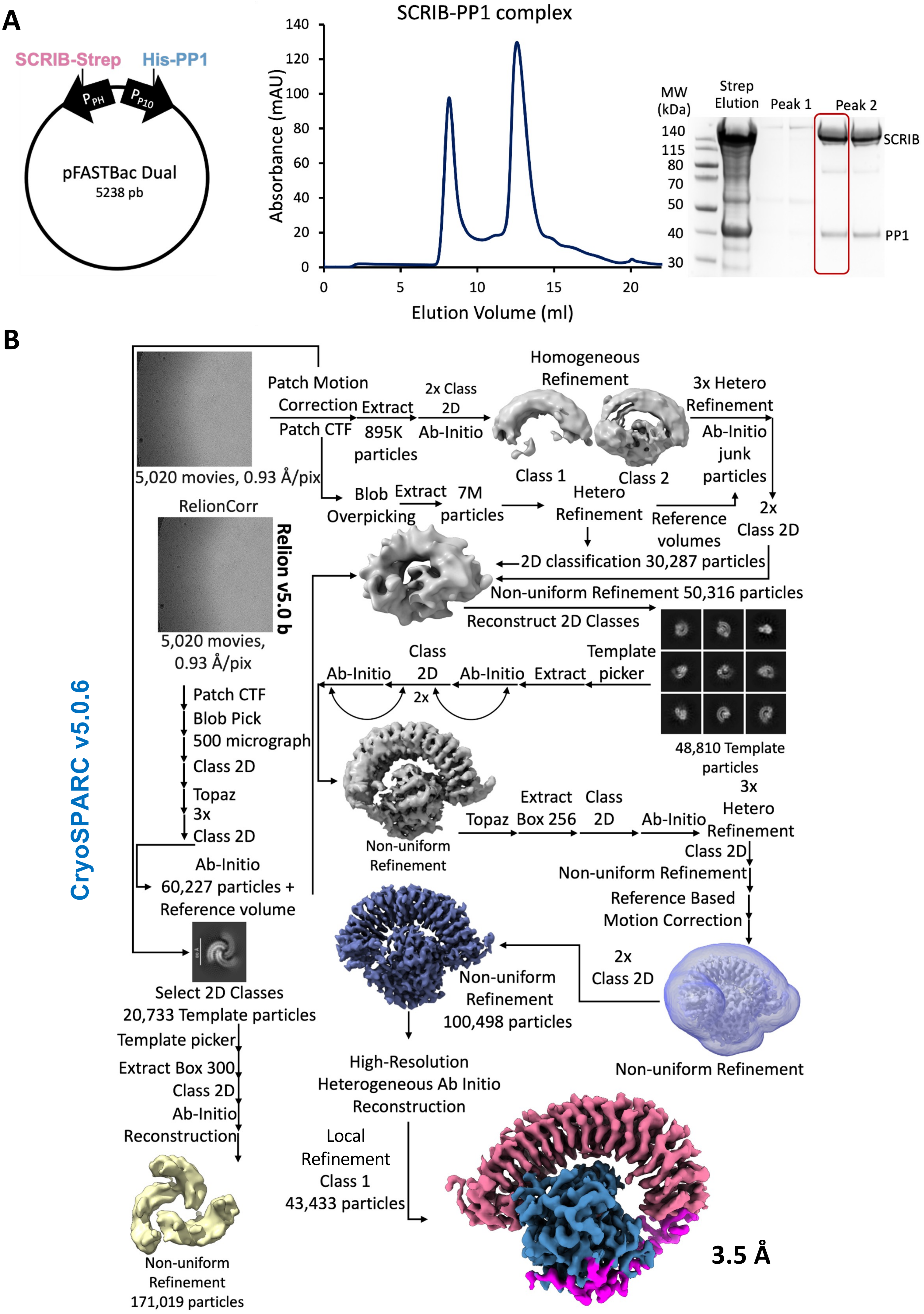

A
SCRIB-Strep
His-PP1
P_{PH}
P_{P10}
pFASTBac Dual
5238 pb
SCRIB-PP1 complex
Absorbance (mAU)
Elution Volume (ml)
MW (kDa)
Strep Elution
Peak 1
Peak 2
140
115
80
70
50
40
30
SCRIB
PP1
B
CryoSPARC v5.0.6
Relion v5.0 b
5,020 movies, 0.93 Å/pix
Patch Motion Correction
Patch CTF
Extract
895K particles
2x Class 2D
Ab-Initio
Homogeneous Refinement
Class 1
Class 2
3x Hetero Refinement
Ab-Initio junk particles
Blob Overpicking
Extract
7M particles
Hetero Refinement
Reference volumes
2x Class 2D
2D classification 30,287 particles
Non-uniform Refinement 50,316 particles
Reconstruct 2D Classes
RelionCorr
5,020 movies, 0.93 Å/pix
Template picker
Extract
Ab-Initio
Class 2D
2x
48,810 Template particles
Patch CTF
Blob Pick
500 micrograph
Class 2D
Topaz
3x
Class 2D
Ab-Initio
60,227 particles + Reference volume
Non-uniform Refinement
Topaz
Extract Box 256
Class 2D
3x Hetero Refinement
Class 2D
Non-uniform Refinement
Reference Based Motion Correction
Select 2D Classes
20,733 Template particles
Template picker
Extract Box 300
Class 2D
Ab-Initio Reconstruction
Non-uniform Refinement 100,498 particles
2x Class 2D
Non-uniform Refinement
High-Resolution Heterogeneous Ab Initio Reconstruction
Local Refinement Class 1 43,433 particles
Non-uniform Refinement 171,019 particles
3.5 Å

Supplementary Figure 1. Biochemical and structural characterization of the SCRIB–PP1 complex. A) Construct design of the pFastBac Dual expression vector with SCRIB and PP1 expressed under the P_{PH} and PP_{10} promoters, respectively. Shown alongside are SEC profiles of the co-purified SCRIB–PP1 complex and the corresponding Coomassie-stained SDS–PAGE gel. **B)** Cryo-EM image processing workflow for the SCRIB–PP1 complex. The diagram also includes representative 2D class averages highlighting self-association of the LRR domain

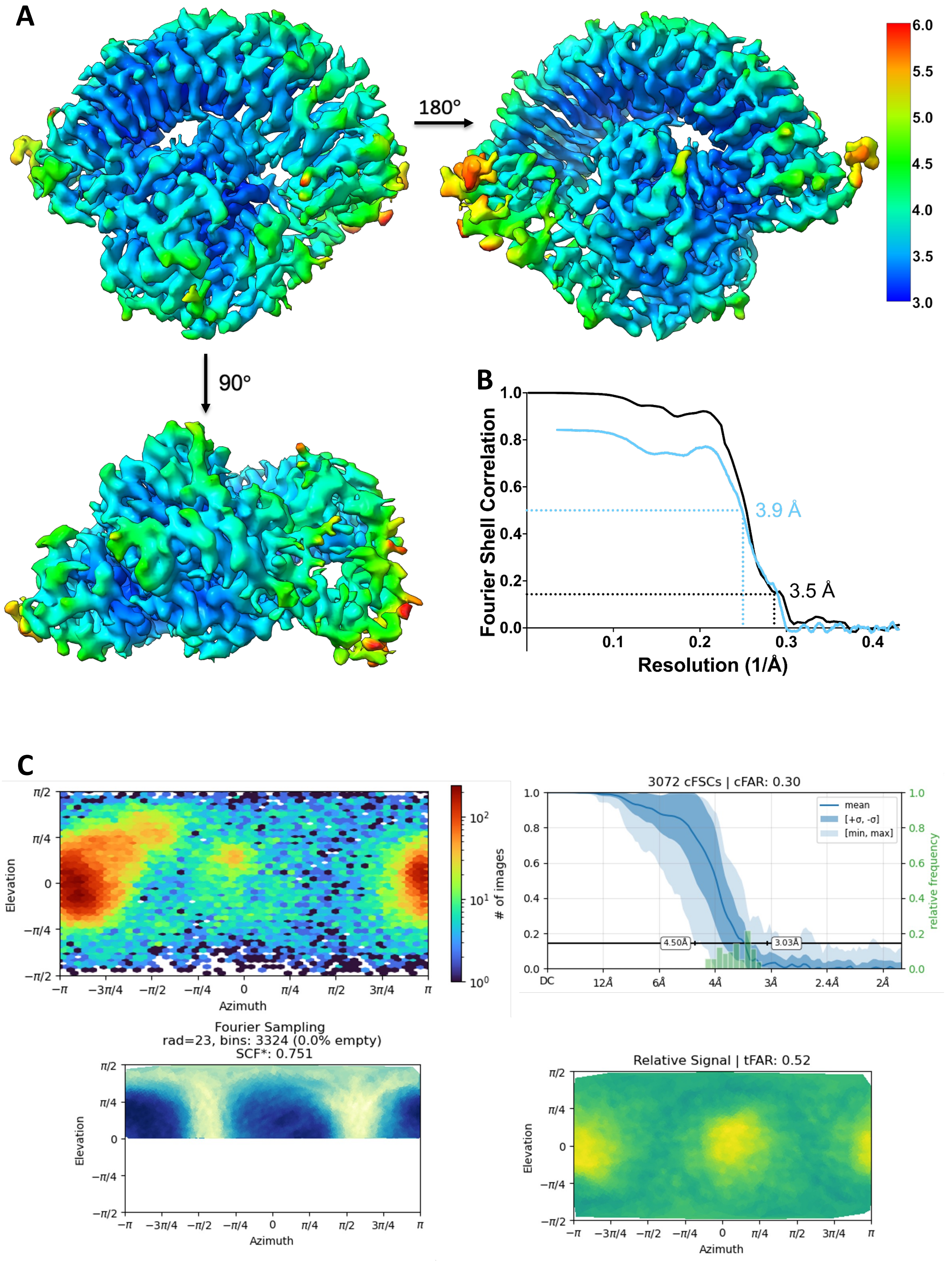
A
180°
90°
6.0
5.5
5.0
4.5
4.0
3.5
3.0
B
Fourier Shell Correlation
Resolution (1/Å)
3.9 Å
3.5 Å
C
Elevation
Azimuth
# of images
3072 cFSCs | cFAR: 0.30
mean
[+σ, -σ]
[min, max]
relative frequency
4.50Å
3.03Å
Fourier Sampling
rad=23, bins: 3324 (0.0% empty)
SCF*: 0.751
Relative Signal | tFAR: 0.52

Supplementary Figure 2. Cryo-EM reconstruction validation metrics of SCRIB-PP1. A) Cryo-EM density map colored by local resolution, with low- to high-resolution regions shown in. blue to red. **B**) Fourier shell correlation (FSC) curves for the cryo-EM reconstructions. Black line represents the correlation between independently processed half maps, with resolution determined at FSC = 0.143. Blue line shows the correlation between the atomic model (PDB 9WMX) and the cryo-EM map (EMD-66108), with the resolution assessed at FSC = 0.5. **C)** Orientation distribution plots of the final reconstruction refined with C1 symmetry. Euler angle (top left), conical FSC (cFSC; top right), sampling compensation factor (SCF; bottom left), and toroidal FSC (tFSC; bottom left) plots are provided.

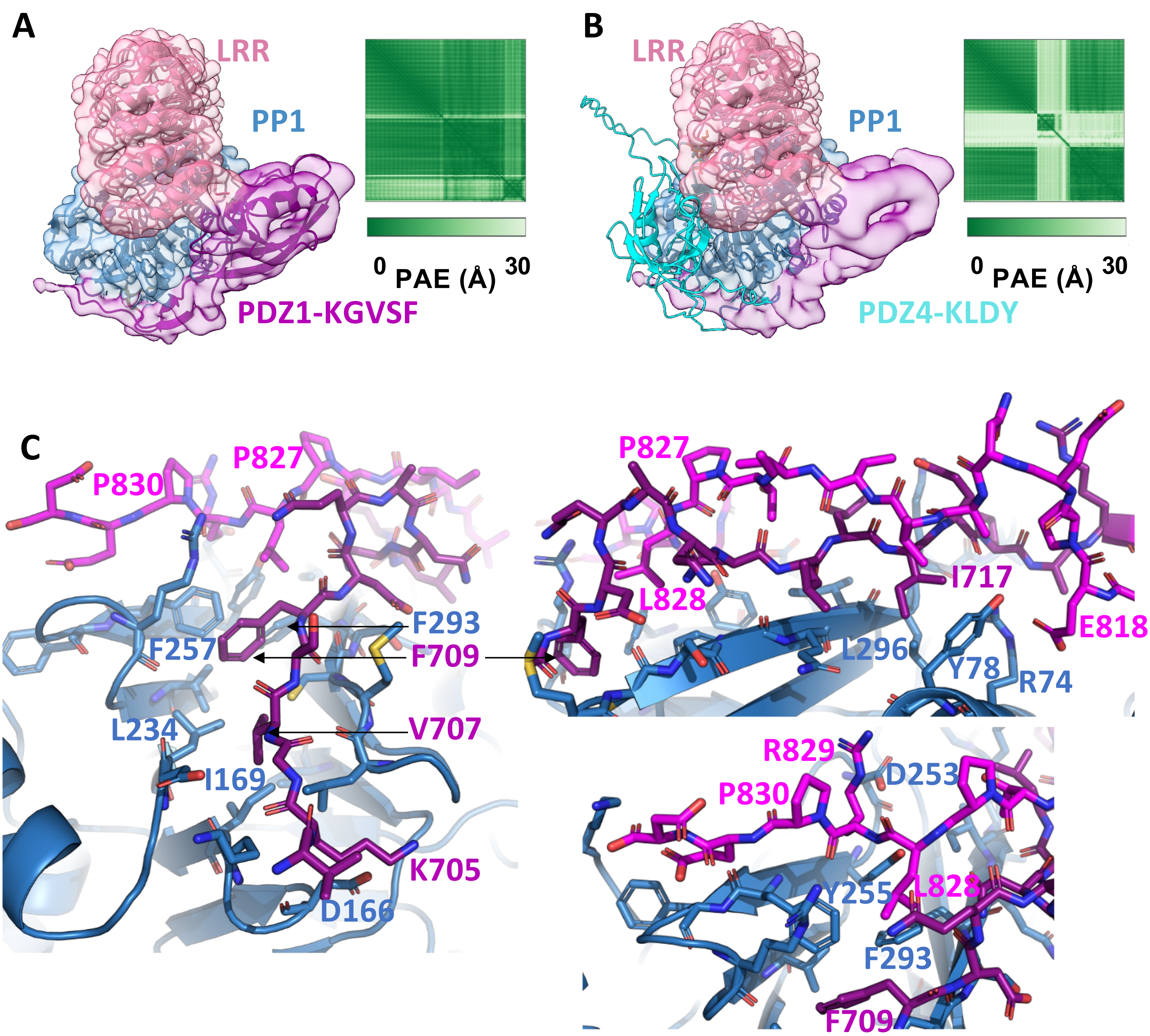

Supplementary Figure 3. Analysis of SCRIB association with PP1. A, B) AlphaFold models of PP1 bound to the SCRIB LRR together with either KGVSF–PDZ1 or PDZ4–KLDY. Predicted aligned error (PAE) plots are shown. **C)** The RVxF pocket of PP1 engages the SCRIB KGVSF motif. This interaction is reinforced by residues C-terminal to PDZ1. The three different orientations highlighting different interaction zones as key interacting residues are labeled. The KVGSF motif and residues N-terminal to PDZ1 are shown in deep purple. C-terminal residues to PDZ1 are shown in magenta. PP1 is shown in blue. Prolines are labelled as structural reference for each orientation.

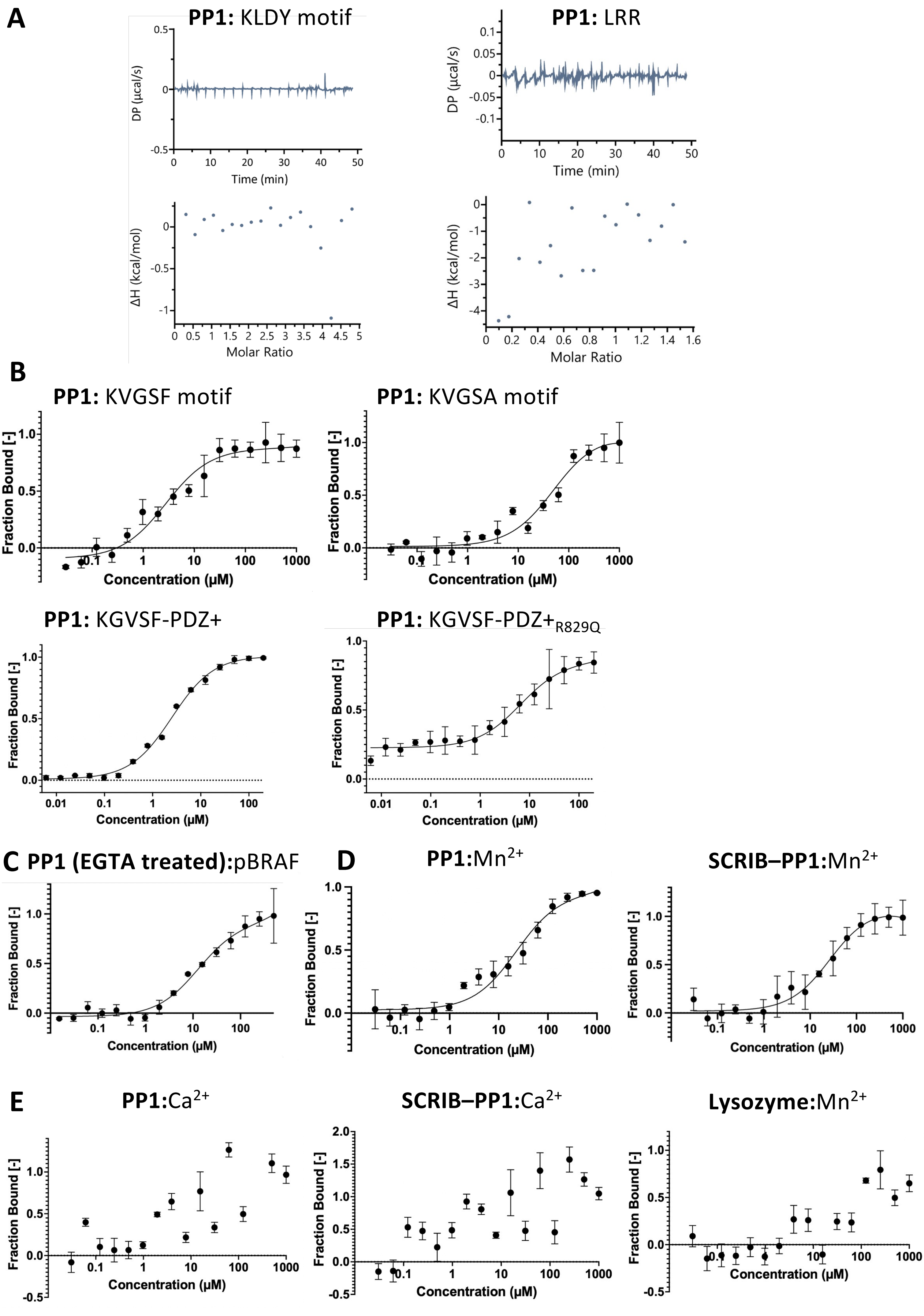

A
PP1: KLDY motif
DP (µcal/s)
Time (min)
ΔH (kcal/mol)
Molar Ratio
PP1: LRR
DP (µcal/s)
Time (min)
ΔH (kcal/mol)
Molar Ratio
B
PP1: KVGSF motif
PP1: KVGSA motif
PP1: KGVSF-PDZ+
PP1: KGVSF-PDZ+R829Q
Fraction Bound [-]
Concentration (µM)
C PP1 (EGTA treated):pBRAF
D
PP1:Mn2+
SCRIB–PP1:Mn2+
E
PP1:Ca2+
SCRIB–PP1:Ca2+
Lysozyme:Mn2+
Fraction Bound [-]
Concentration (µM)

Supplementary Figure 4. Analysis of SCRIB association with PP1. A) ITC profiles of the KLDY motif (left) and LRR (right) titrated against PP1, indicating lack of binding. **B)** Microscale thermophoresis (MST) binding curves for fluorescently labelled PP1 titrated against SCRIB-derived peptides. PP1 bound the KVGSF peptide (K_d = 2.8 ± 1.6 µM), the KVGSA peptide (K_d = 60.7 ± 7.1 µM), the KGVSF–PDZ1+ peptide (K_d = 2.5 ± 0.2 µM), and the KGVSF–PDZ1+ (R829Q) mutant (K_d = 6.5 ± 2.1 µM) (see also **Table 1**). **C)** MST analysis of PP1 interaction with a phosphorylated BRAF peptide (pBRAF) after EGTA treatment to chelate Mn^{2+}. PP1 retains robust binding to pBRAF in the absence of metal (K_d = 12.7 ± 2.0 µM), indicating that substrate recognition is not strictly dependent on the catalytic Mn^{2+} ions. **D)** MST binding curves monitoring Mn^{2+} association with PP1 and the SCRIB–PP1 complex. Both PP1 alone and SCRIB–PP1 exhibit Mn^{2+} binding, with apparent K_d values of 18.8 ± 5.2 µM and 27.2 ± 8.8 µM, respectively, demonstrating that SCRIB association does not prevent occupancy of the catalytic Mn^{2+} pair. Fraction bound was fitted to a one-site binding isotherm to obtain apparent dissociation constants. **E)** MST binding curves monitoring Ca^{2+} association showing no detectable binding of Ca^{2+} to either PP1 or SCRIB–PP1, and no binding of Mn^{2+} to lysozyme.

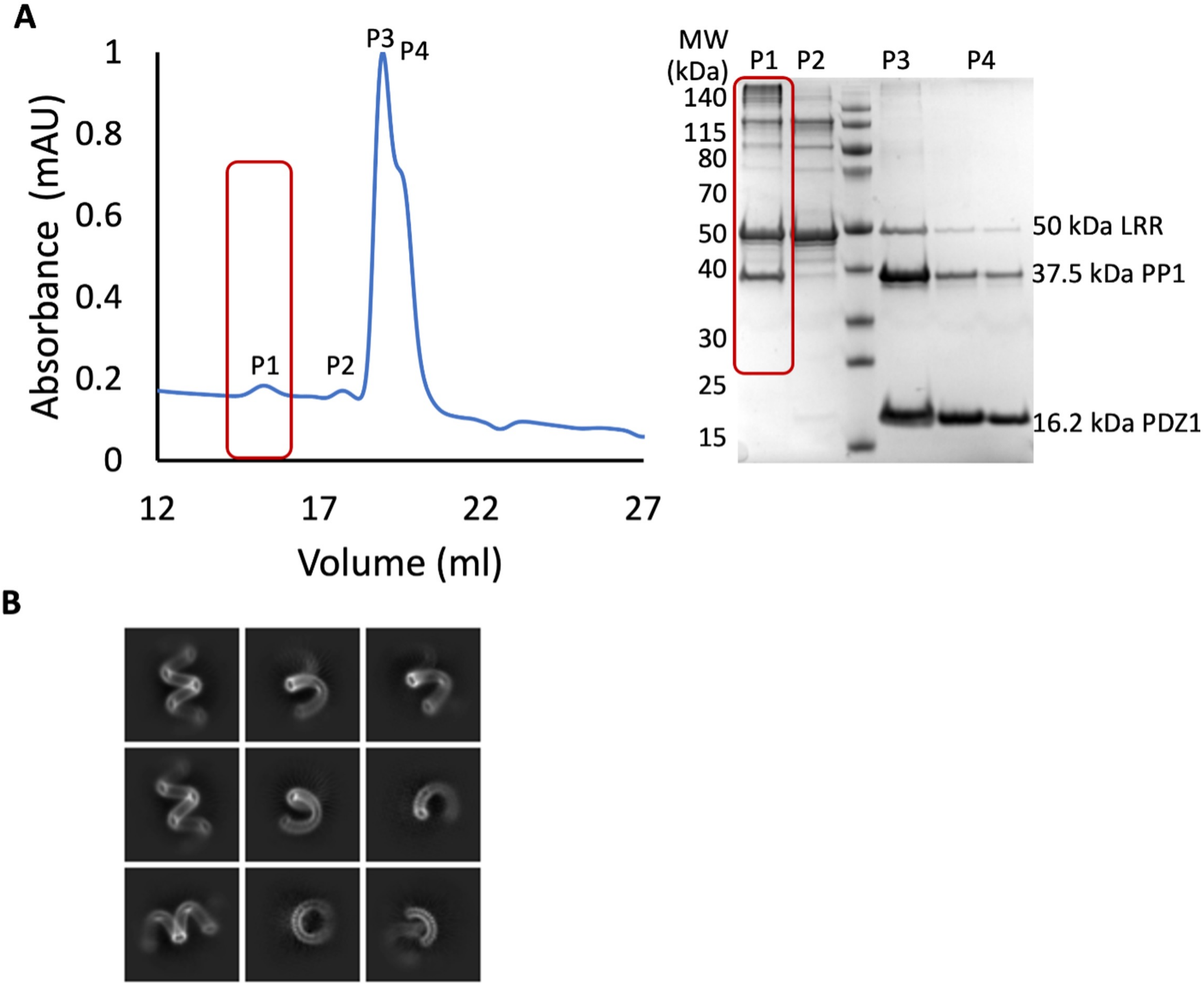

Supplementary Figure 5. Evidence for SCRIB LRR self-association and AlphaFold prediction of SCRIB-mediated PP1 inhibition. A) SEC profile of pre-incubated PP1, LRR, and KGVSF–PDZ1 fragments in a 1:1:2 ratio, showing weak complex formation, with corresponding Coomassie-stained SDS–PAGE gel. **B)** Representative 2D classification averages of peak one, highlighting a tendency for SCRIB LRR multimerization. **C)** AlphaFold model of SCRIB (residues 1–826) in complex with full-length PP1, showing the flexible linker between the LRR and PDZ1 folding back to occlude the PP1 active site.

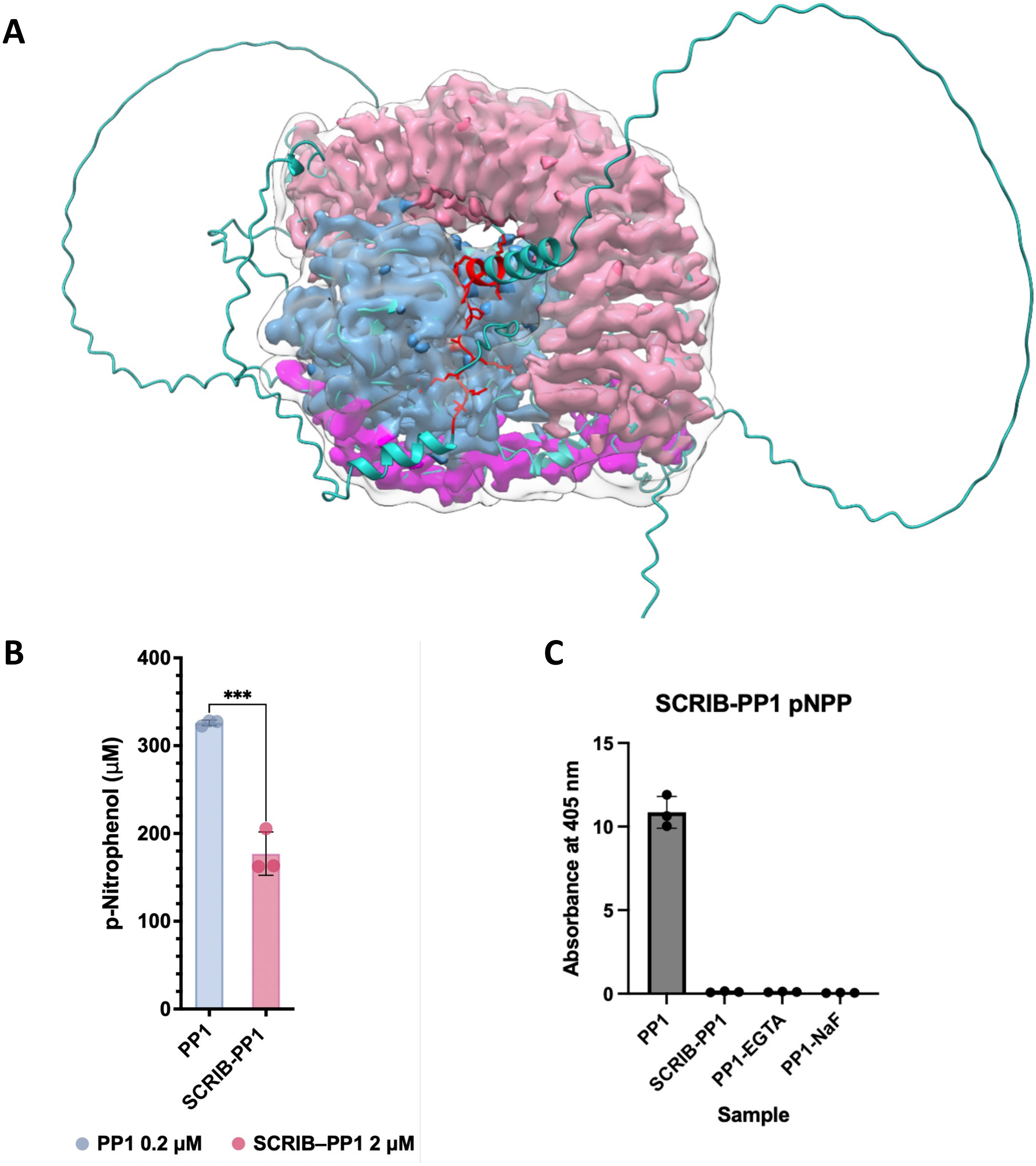

Supplementary Figure 6. Analysis of SCRIB association with PP1. A) AlphaFold model of SCRIB (residues 1–826) in complex with full-length PP1, illustrating the possibility that a region of the flexible linker between the LRR and PDZ1 is folding back toward the active site. The LRR–PDZ1 linker region suggested by AlphaFold is coloured in cyan, with the linker residues suggested to bind to the PP1 core coloured in red. The AlphaFold model is fitted into cryo-EM maps, which are coloured as in Figure 1B, with a low-threshold, B-factor–blurred EM map is shown as a transparent gray surface overlaid on the sharpened reconstruction. **B)** pNPP dephosphorylation assay comparing PP1 alone with a tenfold higher concentration of the SCRIB–PP1 complex, showing persistently reduced catalytic activity. Error bars represent mean ± SD of replicate measurements. **C)** pNPP dephosphorylation assay comparing PP1, the SCRIB–PP1 complex, and PP1 pre-treated with EGTA or NaF at a protein concentration of 500 nM. PP1 displays strong phosphatase activity, whereas the SCRIB–PP1 complex and inhibitor-treated PP1 show negligible turnover, confirming that SCRIB binding functionally suppresses PP1 catalytic activity.

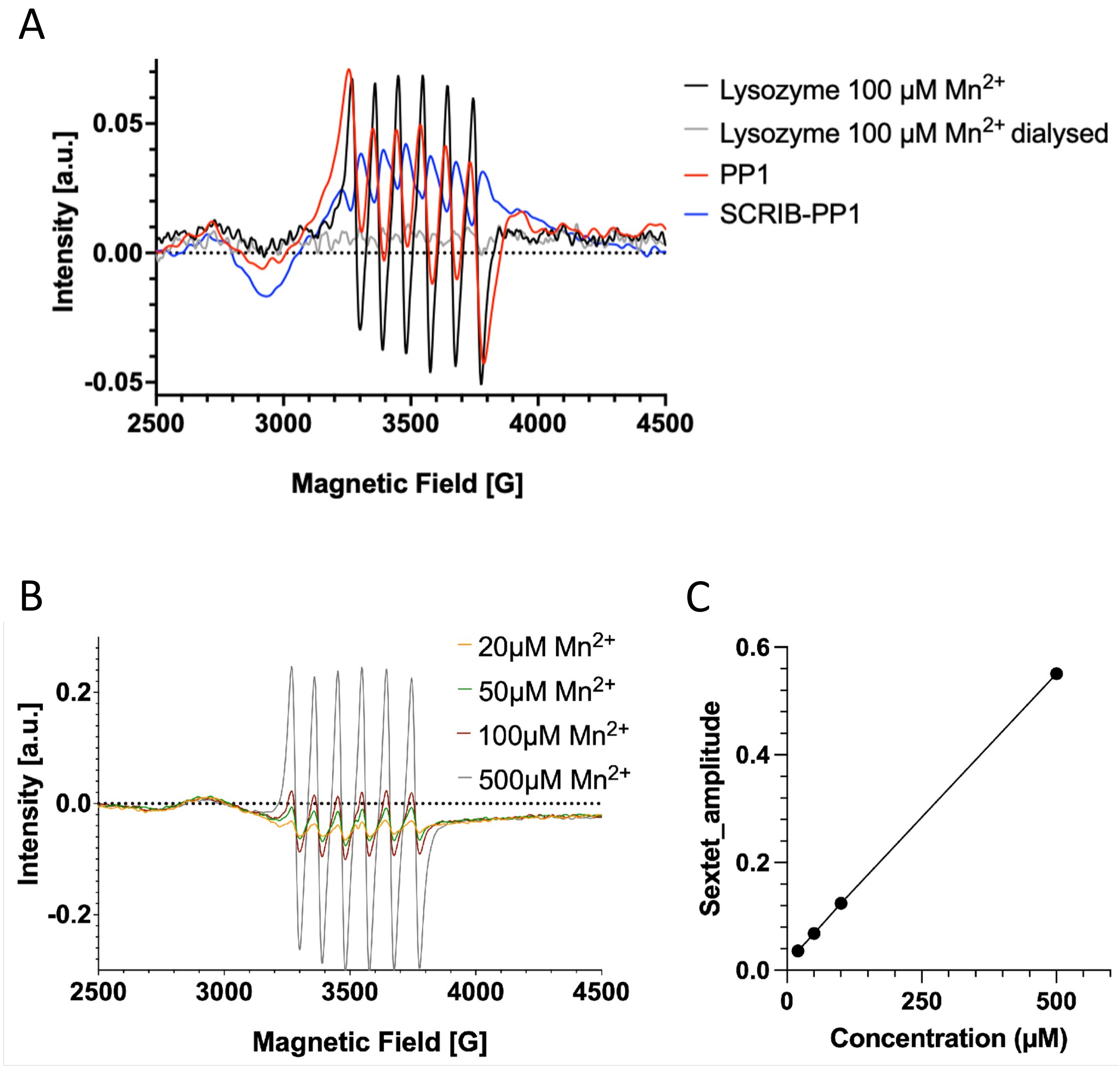

Supplementary Figure 7. EPR-based quantification of Mn^{2+} binding to PP1 and the SCRIB-PP1 complex. ***(A)*** Continuous-wave X-band EPR spectra of Mn^{2+} in different sample conditions. Free Mn^{2+} (maroon) and lysozyme + Mn^{2+} (black) exhibit sharp, well-resolved sextets characteristic of aqueous Mn^{2+}. Dialysed lysozyme (grey) shows only a weak residual signal, defining the non-specific Mn^{2+} background. In contrast, PP1 (red) and the SCRIB-PP1 (blue) retain substantially stronger and broader sextets following dialysis, consistent with protein-bound Mn^{2+}. ***(B)*****CW-EPR spectra of $MnCl_2$ standards.** Room-temperature X-band CW-EPR spectra of four free $MnCl_2$ standards recorded in EPR buffer (50 mM HEPES pH 7.4, 150 mM NaCl, 1 mM DTT). Concentrations were: 20 μM (orange); 50 μM (green); 100 μM (dark red); 500 μM (grey). All spectra display the characteristic six-line (sextet) hyperfine pattern of high-spin Mn^{2+} (S = 5/2, ^{55}Mn I = 5/2) in the 3300–3800 G region. Signal amplitude scales linearly with Mn^{2+} concentration across the full range. Instrument settings: Bruker EMX, ~9.4 GHz, sweep width 3000 G, modulation amplitude 14 G, 3 scans. Spectra are shown without further processing. The dotted line indicates zero intensity. **(C) Mn^{2+} calibration curve.** Sextet amplitude (peak-to-trough intensity within 3300–3800 G) plotted against $MnCl_2$ concentration for standards. Linear regression yielded: Amplitude = $(1.07 \times 10^{-3}) \times [Mn^{2+}] + 0.0154$, $R^2 = 0.99997$, confirming a linear detector response across 20–500 μM. This calibration curve was used to convert sextet amplitudes of experimental samples to absolute Mn^{2+} concentrations. Data points represent single measurements; the line represents the linear regression fit.

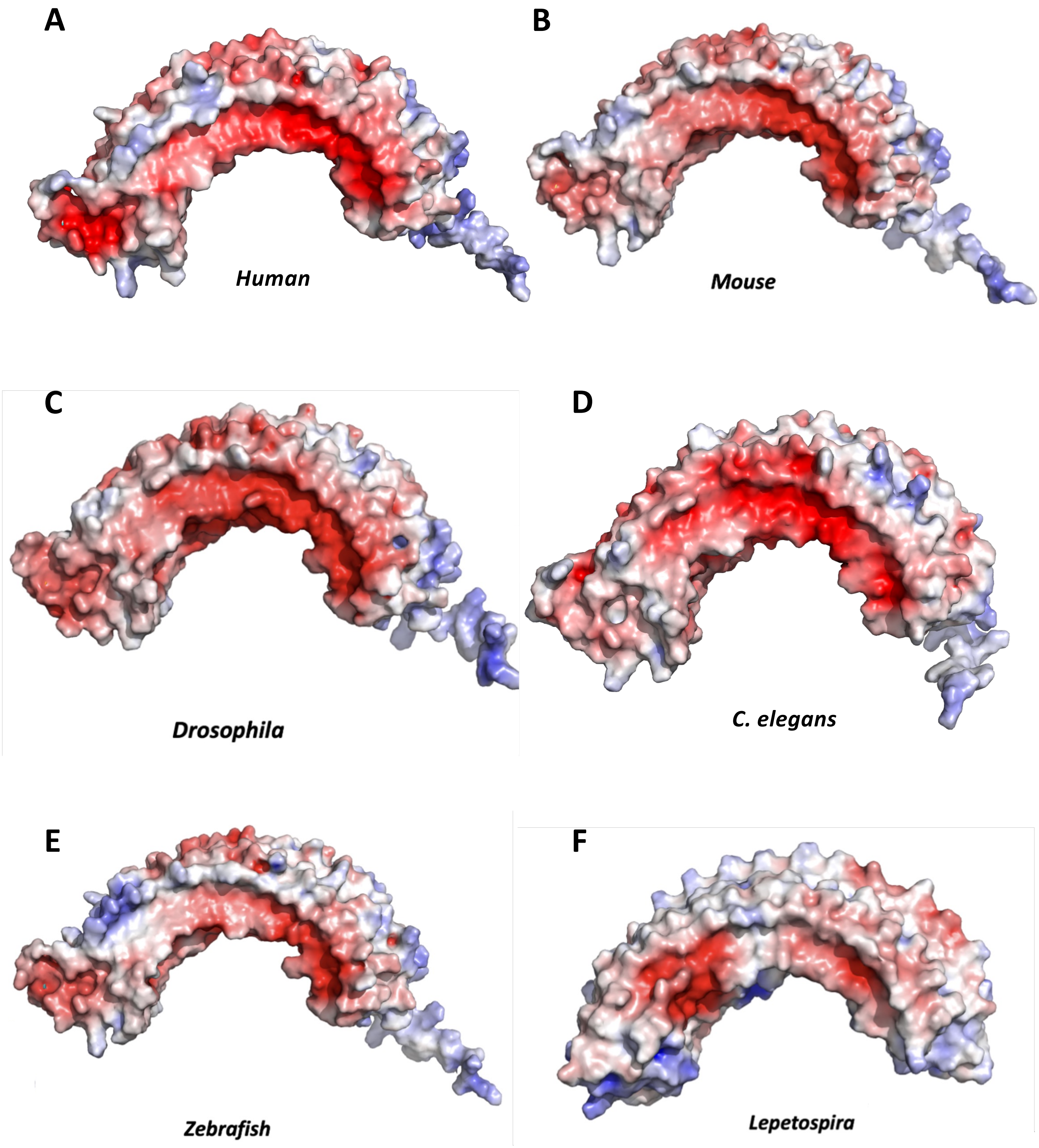

Supplementary Figure 8. Electrostatic surface of conserved SCRIB LRR domains across species. A) *Human* SCRIB LRR domain (from the construct used in this study). **B)** Mouse SCRIB LRR domain (homolog of human SCRIB). **C)** *Drosophila* SCRIB LRR domain (homolog of human SCRIB). **D)** *C. elegans* SCRIB LRR domain (homolog of human SCRIB). **E)** *Zebrafish* SCRIB LRR domain (homolog of human SCRIB). **F)** LRR protein from *Leptospira* (bacterial LRR; uniprot ID: M6RQD1). Unlike eukaryotic SCRIB, bacteria do not encode proteins harboring both LRR and PDZ domains.

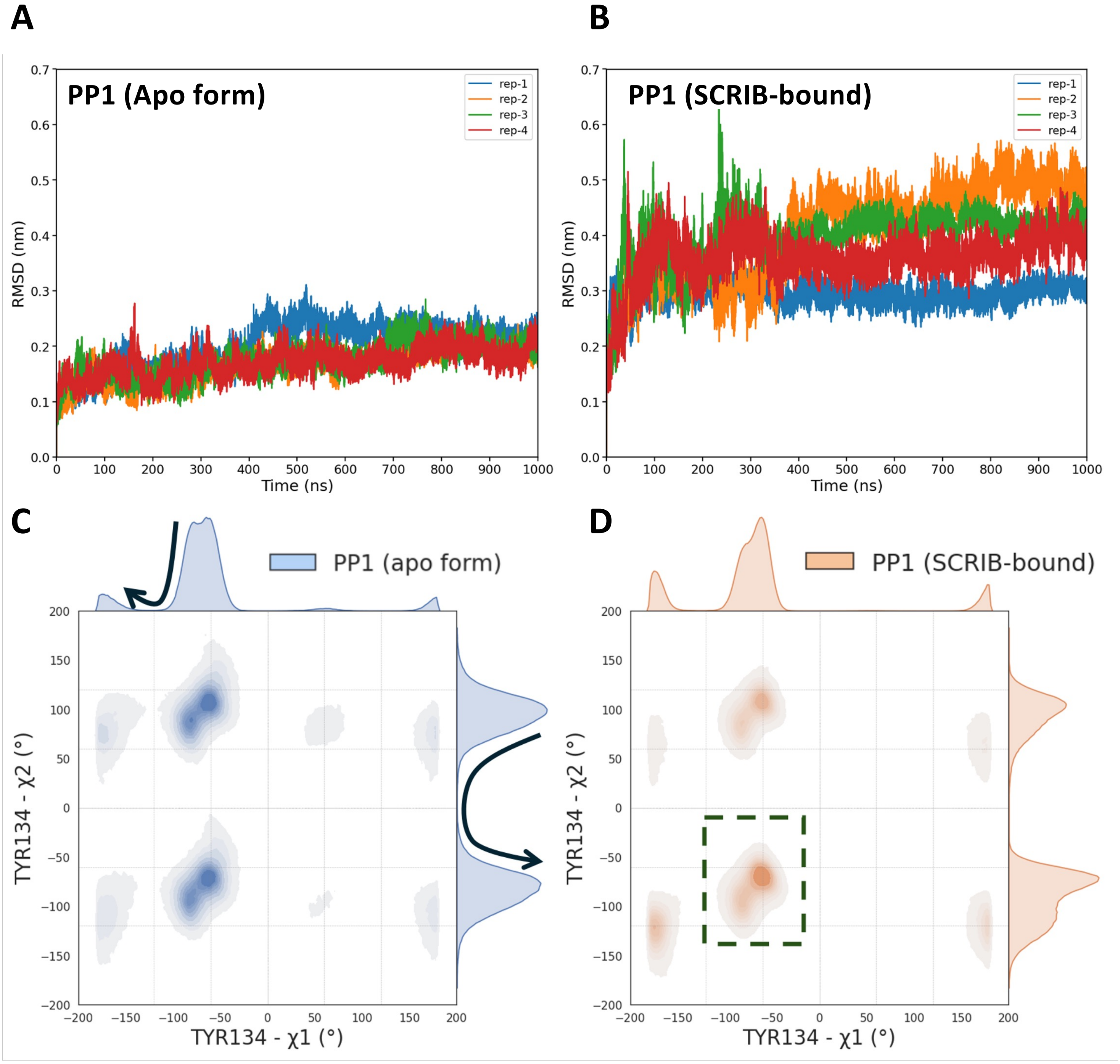

Supplementary Figure 9. Backbone root mean square deviation (RMSD) for (**A**) PP1 (apo form) and (**B**) PP1 (SCRIB-bound) complex. The RMSD is calculated after aligning onto the minimized starting structure for each system. All systems remained stable and well equilibrated throughout the 1 µs production runs across four independent replicas. The complex exhibited higher RMSD values with increased fluctuations in PDZ1 domain in certain replicas. (**C**, **D**) Chi (χ) dihedral angle distributions of residue Tyr134 in PP1, which exhibited increased fluctuations in the RMSF analysis (Figure 6). The color opacity indicates the population density within each bin. The figure integrates two-dimensional distributions (χ1 vs. χ2) with one-dimensional marginal histograms, illustrating population shifts in dihedral angles upon complex formation. Arrows indicate directional shifts in the dihedral distribution for PP1 in complex with LRR and PDZ1 domains, and the dashed square highlights the most populated conformational basin in the complex state.

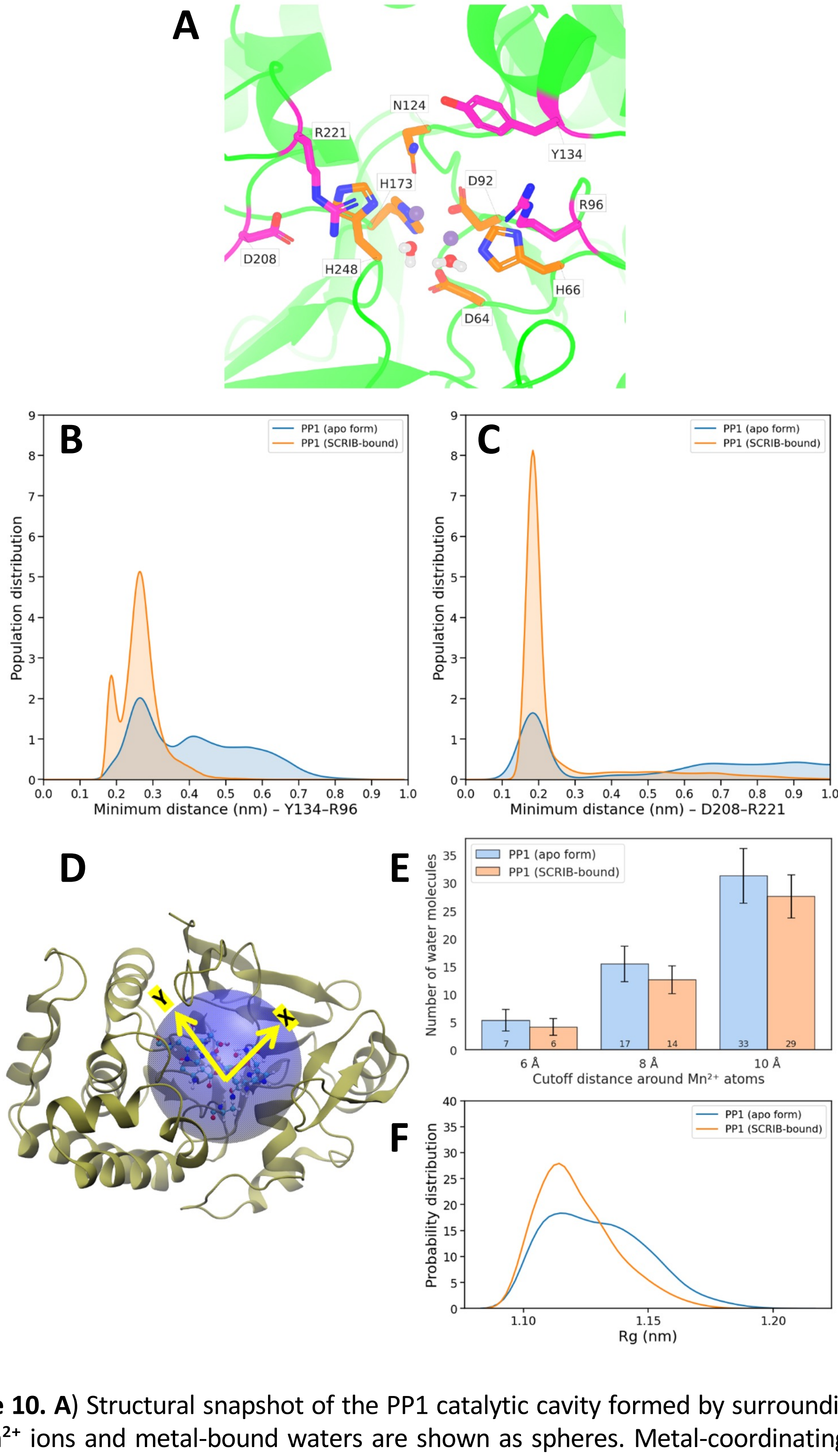

Supplementary Figure 10. A) Structural snapshot of the PP1 catalytic cavity formed by surrounding loops (green cartoon). The two Mn^{2+} ions and metal-bound waters are shown as spheres. Metal-coordinating residues (e.g., Asp64, His66, Asp92, N124, His173, His248) are shown as orange sticks; nearby charged/polar residues analyzed in panels B–C are highlighted and labeled. (B–C) Minimum distance distribution between Tyr134–Arg96 (**B**) and Asp208–Arg221 (**C**), comparing apo PP1 (blue) and SCRIB-bound PP1 (orange). In both pairs, SCRIB binding shifts the population toward shorter, unimodal distribution that stabilizes the local interactions near the catalytic center. (D-F) Structural and hydration changes around the PP1 metal-binding center upon SCRIB-PDZ1 binding. (**D**) PP1 structure highlighting the Mn^{2+} ions surrounded by coordinating residues. The blue sphere illustrates the area within 12 Å of these ions. Axes X and Y represent directional vectors for reference. (**E**) Number of water molecules within specified radial distances (6, 8, and 10 Å) around the Mn^{2+} ions, averaged over all the replicas. A subtle decrease in water occupancy near the metal center is observed in complex compared to the apo state. (**F**) Radius of gyration (Rg) distribution calculated for residues within 12 Å of the Mn^{2+} ions over all replicas. The broader distribution in the apo form suggests increased structural flexibility, while the narrower distribution in the complex implies a modestly constrained metal-binding environment. These subtle structural and hydration alterations, while not dramatic, could contribute to functional modulation of PP1 upon binding to SCRIB.

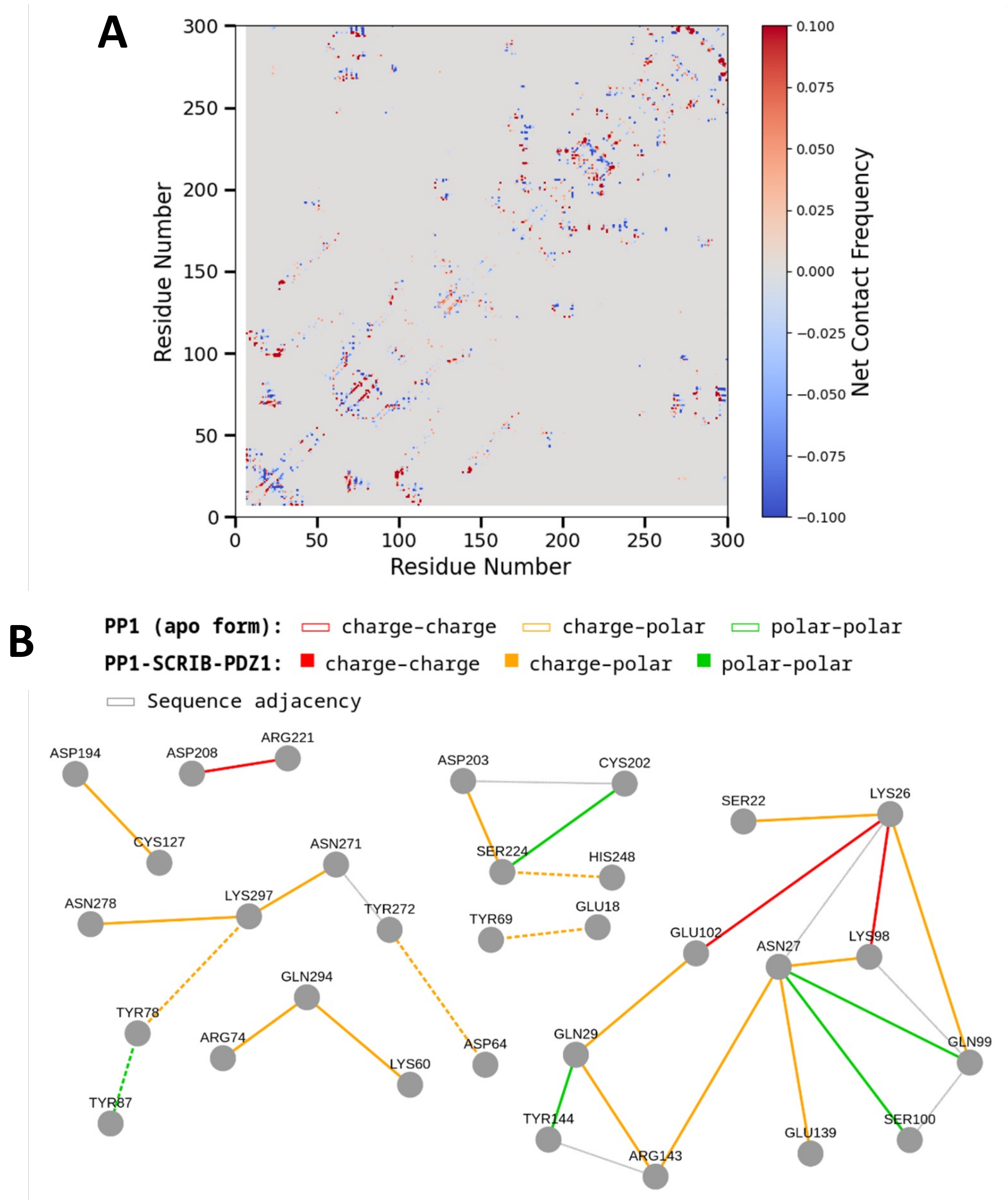

Supplementary Figure 11. A) Differential contact frequency map for PP1, highlighting residue-residue contacts with altered frequency between the apo form and the complex. This map is generated by subtracting contact frequencies in the apo simulations from those in the complex, with values ranging from −1 (contacts exclusive to apo) to +1 (contacts exclusive to complex). Blue regions represent contacts that are more frequent in the apo state, while red regions indicate contacts that are more frequent in the complex. The differential contact frequency values for residue pairs are provided in Table S1. **B)** Network representation of PP1 intra-protein contacts in the apo form (dashed edges) and in complex form (solid edges). Only contacts between charged or polar residues (gray nodes) with a net contact frequency greater than 0.25 are shown: red edges denote charge–charge, orange edges charge–polar, and green edges polar–polar interactions. Light-gray lines connect sequentially adjacent residues. Table S1 provides a comprehensive, categorical listing of all contacts involving charged, polar, and hydrophobic residues.

A

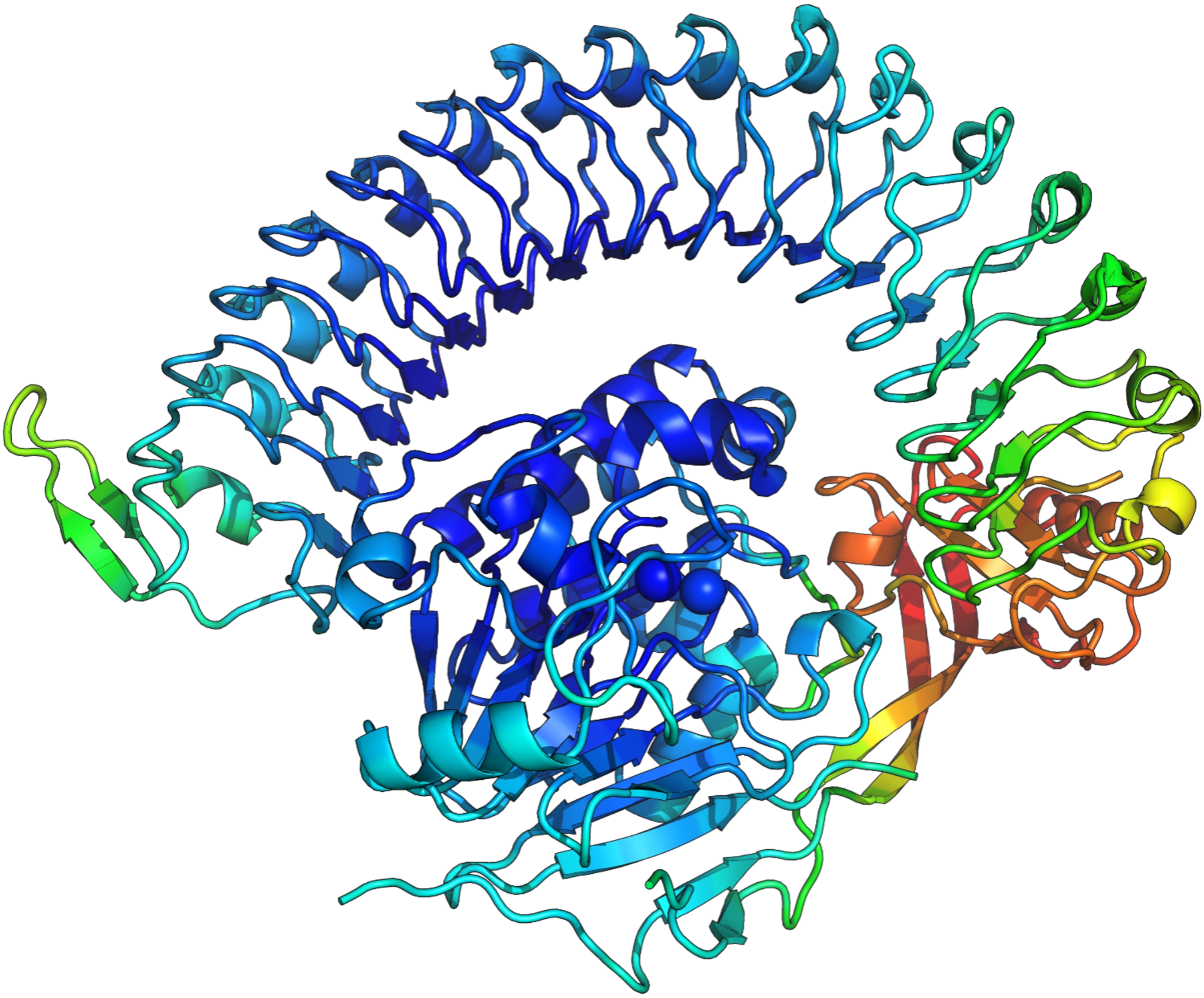

B

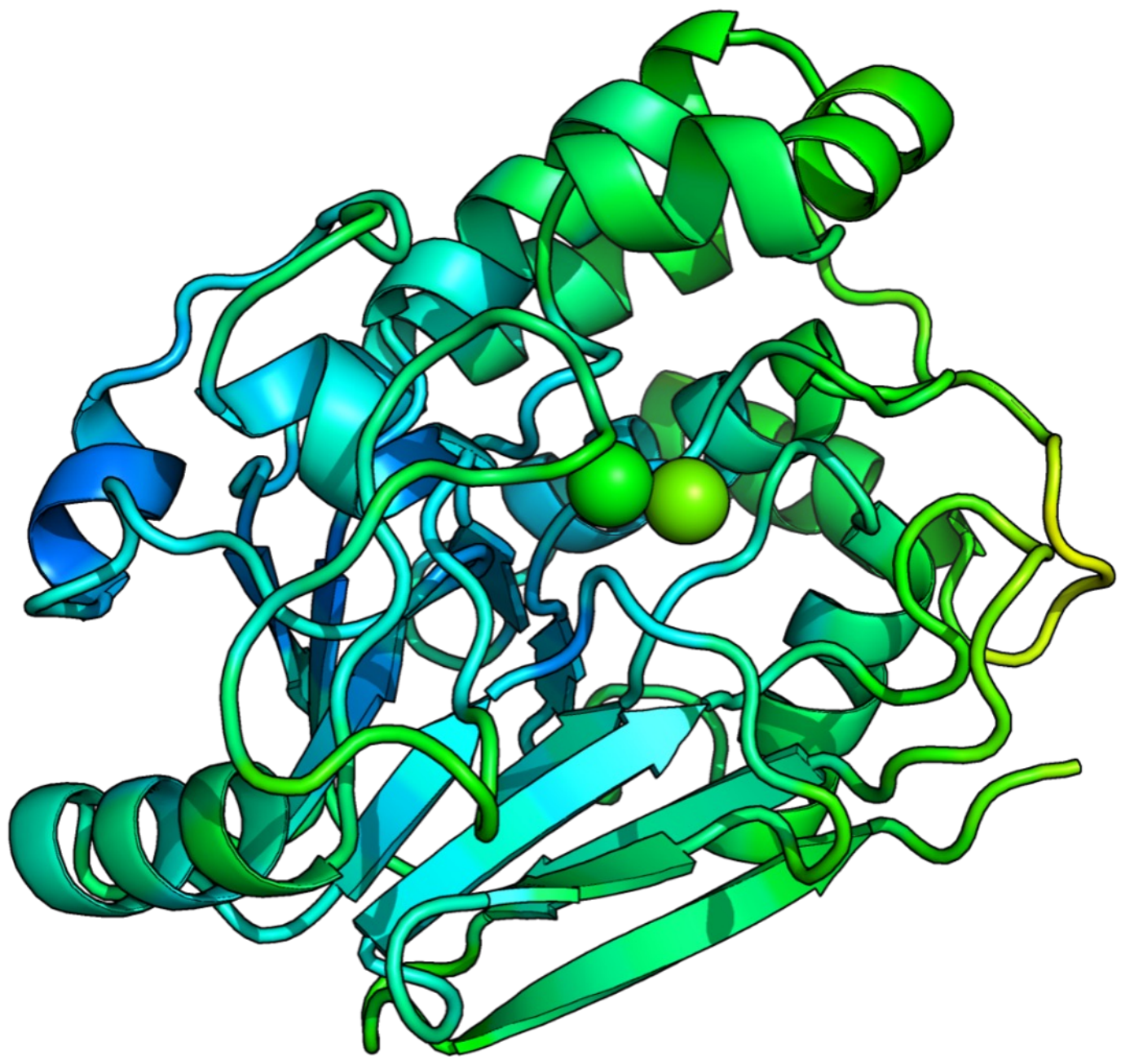

Supplementary Figure 12. B-factor analysis of the SCRIB–PP1 cryo-EM structure. A) B-factor colorings of the cryo-EM structure highlight that the LRR, and to some extend the KGVSF region, stabilize PP1. **B)** B-factor coloring of active PP1 in complex with SHOC2 and MRAS (PDB 7UPI) shown for comparison (SHOC2 and MRas not shown)

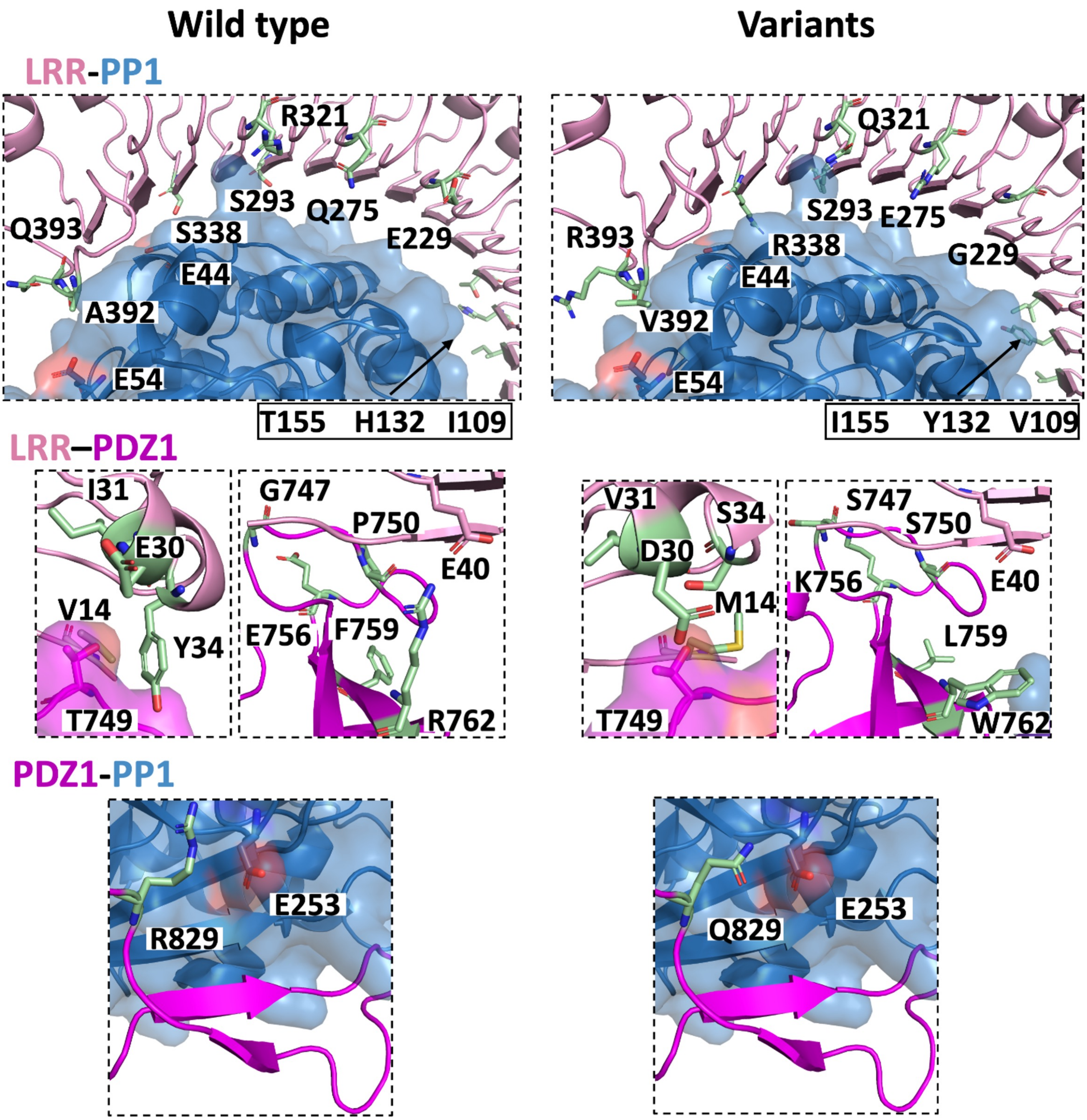

Supplementary Figure 13. ClinVar-reported SCRIB variants of uncertain significance (VUS) mapped on the SCRIB–PP1 complex. LRR variants disrupt electrostatics (E229G, Q275R, R321Q) or alter hydrogen bonding (S338R, Q393R), while E30D and I31V destabilize the first LRR motif. LRR–PDZ1 interface variants (V14M, Y34S) weaken interdomain contacts, and PDZ1 variants (G747S, P750S, E756K, F759L, R762W) disrupts local loop dynamics or electrostatic interactions. R829Q in the PDZ1 extension may further disrupt PP1 binding.

A

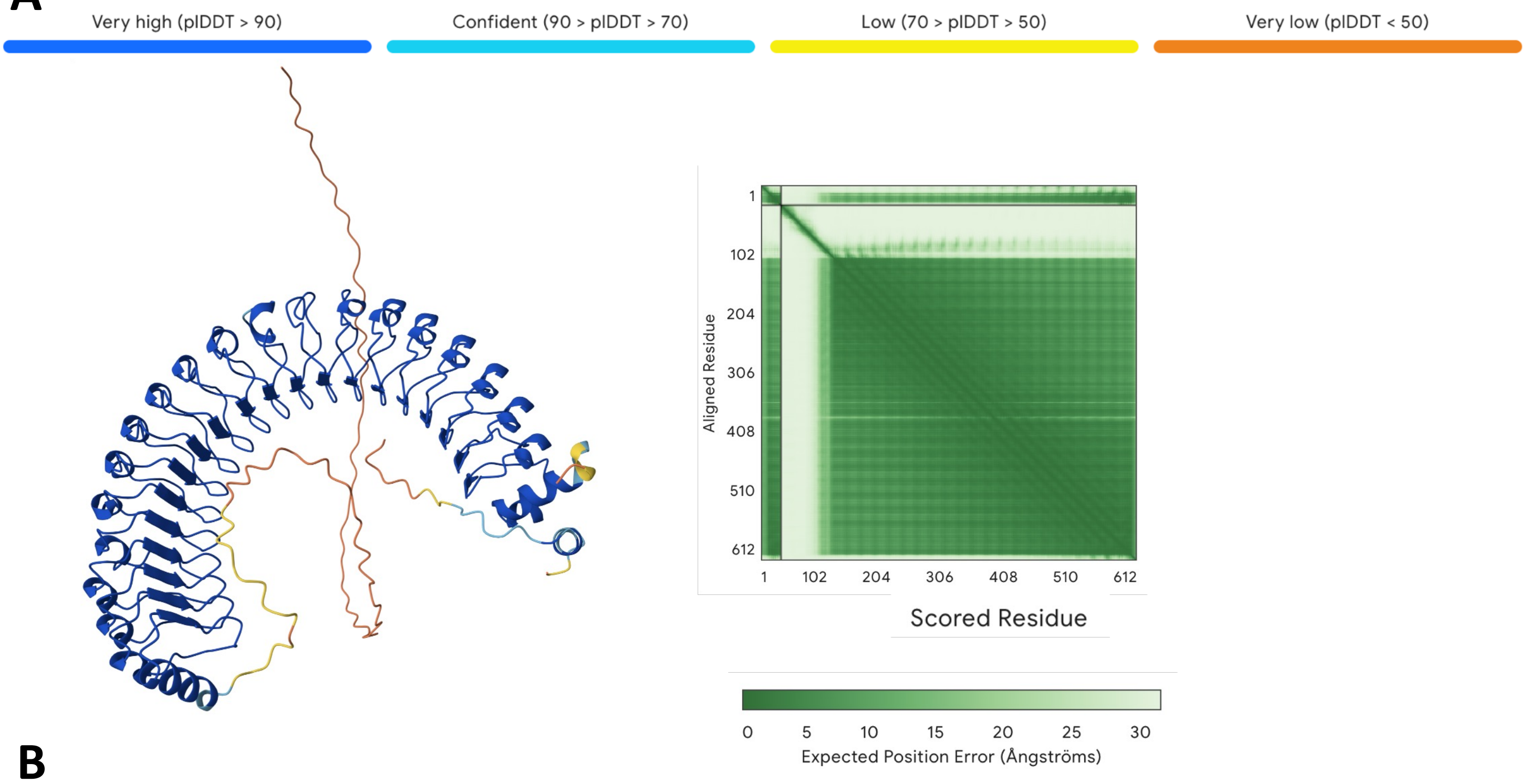

B

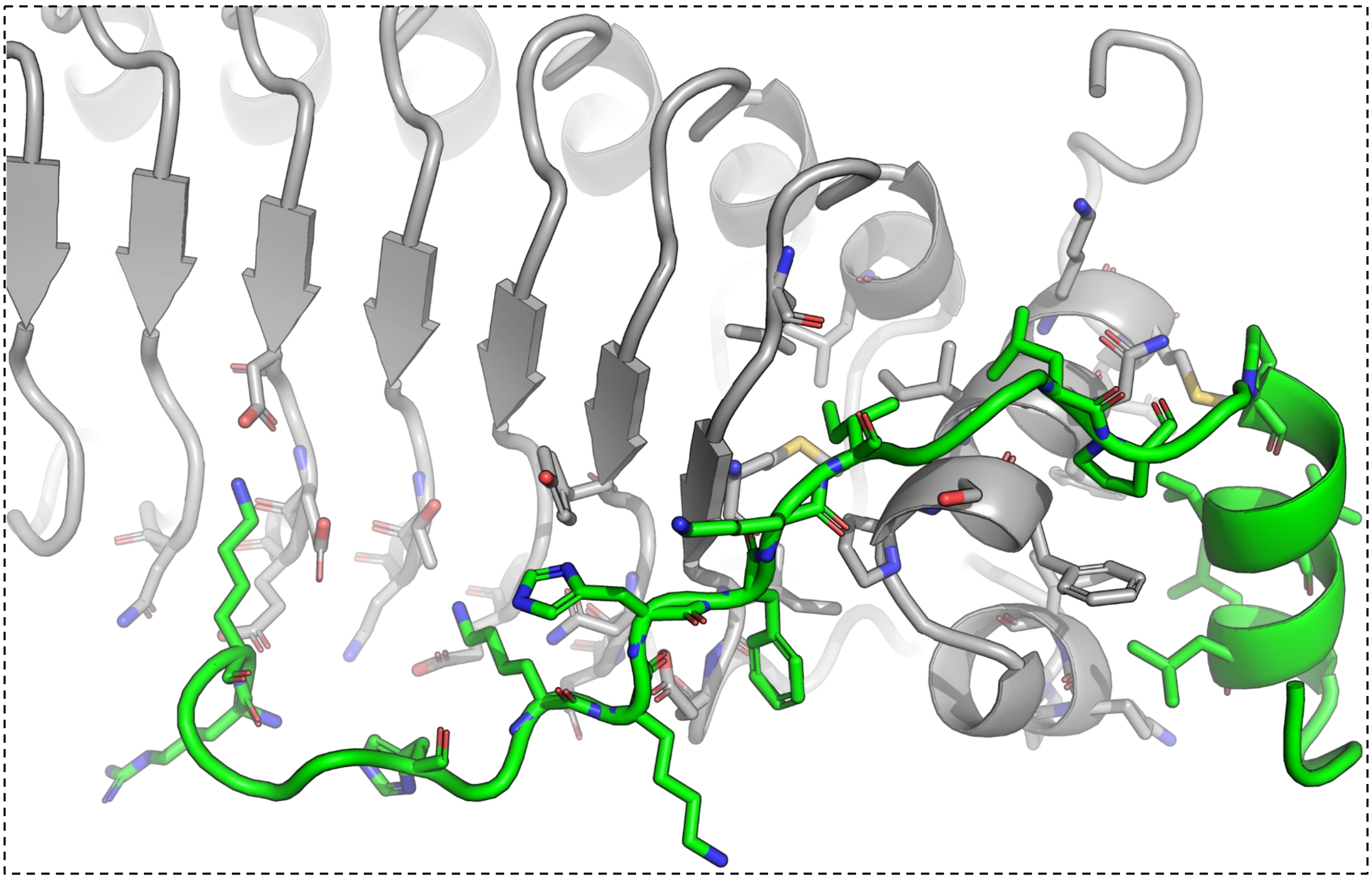

Supplementary Figure 14. AlphaFold prediction of the SCRIB–SHOC2 interaction. A) AlphaFold model with pLDDT and PAE plots predicts that the linker between the SCRIB LRR and PDZ1 engages the C-terminal LRR of SHOC2. **B)** SCRIB residues 614–634 form a loop–helix element that hydrophobically associates with SHOC2's C-terminal helices, while residues 604–613 may form electrostatic interactions with its convex surface, consistent with (Mol Cell Young, L. C. *et al*. 2013).

	**SCRIB-PP1**<br>EMD-66672<br>PDB 9X9Y
**Data collection and processing**	
Magnification	130k
Voltage (kV)	300
Electron exposure (e^{-}/Å^{2})	36.4
Defocus range (μm)	-2.5 – -1.6
Pixel size (Å)	0.93
Symmetry imposed	C1
Initial particle images (no.)	661k
Final particle images (no.)	43,433
Map resolution (Å)<br>(FSC threshold 0.143)	3.5
Map resolution range (Å)	3.1 – 8.8
**Refinement**	
Initial model used	AlphaFold
Model resolution (Å)<br>(FSC threshold 0.5)	3.5
Model resolution range (Å)	3.12 – 8.76
Map sharpening *B* factor (Å^{2})	-268.5
Model composition	
Non-hydrogen atoms	6,537
Protein residues	831
Ligands	2
Waters	0
B factors (Å^{2})	
Protein	71.72
Ligand	51.41
Water	28.68
R.m.s. deviations	
Bond lengths (Å)	0.13
Bond angles (°)	0.36
Validation	
MolProbity score	1.64
Clashscore	5.00
Poor rotamers (%)	0.97
Ramachandran plot	
Favored (%)	94.79
Allowed (%)	5.21
Disallowed (%)	0.00

Supplementary Table 1. Cryo-EM data collection, refinement and validation statistics

Parameter	30 µM Lysozyme with 100 µM Mn^{2+}	30 µM Lysozyme with 100 µM Mn^{2+} dialysed	PP1	SCRIB-PP1
**g-value**	2.00 ± 0.01	2.00 ± 0.01	2.00 ± 0.01	2.00 ± 0.01
**A_hf (G)**	86.5 ± 1.5	86.5 ± 1.5	86.5 ± 1.5	86.5 ± 1.5
**Avg. linewidth Bpp (G)**	31 ± 1	43 ± 22	44 ± 6	44 ± 9
**Sextet amplitude (a.u.)**	0.11963	0.01205	0.09235	0.03110
**Amplitude ratio vs control**	100%	10.1%	77.2%	26.2%
**Visual peak height ratio vs control**	100%	~10%	~76%	~28%
**[Total Mn^{2+}] (µM)**	100.7	10.1 (background)	77.7	26.2
**[Net bound Mn^{2+}] (µM)**	N/A	N/A	67.1	16.1
**Protein concentration (µM)**	N/A	N/A	27.5	7.5
**Mn^{2+} / molecule**	N/A	N/A	2.4 ± 0.3	2.2 ± 0.2

Supplementary Table 2. Continuous-wave electron paramagnetic resonance (CW-EPR) parameters for Mn^{2+} determined from X-band microwave frequency range spectra. The Electron g-factor (*g*-value) is fixed at the free-electron value for high-spin Mn^{2+} (d^5, S = 5/2), where d^5 is d^5 electron configuration (five electrons in the d-orbitals) and S is Total electron spin quantum number. Isotropic hyperfine coupling constant (A_hf), in gauss (G) is fixed at the literature value of 86.5 ± 1.5 G for aqueous $^{55}Mn^{2+}$ (I = 5/2, 100% natural abundance). Line centres in PP1 and SCRIB-PP1 could not be independently resolved due to broadening and reduced signal-to-noise. A_hf was measured from lysozyme with Mn^{2+} and Mn^{2+} alone (where lines are sharp) and confirmed consistent with the literature value of 86.5 G before being applied to all samples Linewidth (peak-to-peak linewidth, Bpp) is reported as the mean ± SD of sextet lines 1, 2, 5, and 6. Mn^{2+} concentrations were quantified by the sextet amplitude method (peak-to-trough intensity within 3300–3800 G), calibrated against a four-point $MnCl_2$ standard curve (20–500 µM; R^2 = 0.99997) **(Suppl. Figure 7B)**. Net bound Mn^{2+} was obtained by subtracting the lysozyme dialysed background (10.1 µM). Stoichiometry (Mn^{2+} per molecule) was calculated by dividing net bound Mn^{2+} by the protein concentration determined by A_{280} absorbance. The broader linewidth observed for PP1 and SCRIB–PP1 relative to lysozyme with Mn^{2+} (~44 G vs ~31 G) is consistent with protein-bound Mn^{2+} undergoing restricted rotational tumbling and zero-field splitting contributions from the asymmetric coordination environment. N/A, not applicable.
